# Supplementary material for: Examining the efficacy of localised gemcitabine therapy for the treatment of pancreatic cancer using a hybrid agent-based model
Source: PLoS Comput Biol. 2023 Jan 17;19(1):e1010104. doi: 10.1371/journal.pcbi.1010104 (PMC9891514; doi:10.1371/journal.pcbi.1010104)
Supplement: S1 Text — Supplementary Tables and Figures that support results in the main text. Includes Tables A-E and Figs A-O. (DOCX) [file pcbi.1010104.s001.docx]

**Supplementary Tables and Figures: Examining the efficacy of drug-loaded polymers in the treatment of pancreatic cancer using a hybrid agent-based model.**

Adrianne L. Jenner^1^, Wayne Kelly^2^, Michael Dallaston^1^, Robyn Araujo^1^, Isobelle Parfitt^1^, Dominic Steinitz^3,4^, Pantea Pooladvand^5^, Peter S. Kim^5^, Samantha J Wade^6^, Kara L. Vine^6,7^,

**Table A. Fibre release parameters.** Parameters for the diffusion and release of drug from the fibre $k$, $D_{const}$, $C_{0}$ and $A_{out}$ (see **S1 Technical Supplementary Information** and **Eqs TS10-TS13**) were fit to varying percentages of alginate and PCL. In our simulations, we used the parameters obtained from a 3% alginate and 15% PCL fibre with corresponding fit in **Fig 3**B. The values for other fibre configurations 1% alginate and 2% alginate are provided as a way of validating the model’s ability to match the drug release experiments. The fits for 1% alginate and 2% alginate are in **Fig G.** We fixed $\epsilon=1$ hour in **Eq. (5)**, to obtain a finite initial diffusion coefficient $D_{F}(0)$ and $r_{total}$ to the measured average radius of the fibres [1, 2]. Note, $A_{out}$ is the area of the well in the *in vitro* experiment and was used to approximate the total area the drug flows into $h\times\left( \Delta\times N \right)^{2}$, where $h$ was the height of the rectangular domain (**Fig A**), $\Delta$ was the length of the voxels and $N$ was the number of voxels in the FVM discretisation. We assume this can differ across the experiments as the size of the fibre changes with the introduction of different concentrations of alginate as seen in $r_{total}$.

| **Params Units** | | **Description** | 1% alginate | 2% alginate | 3% alginate 15% PCL | Ref |
| --- | --- | --- | --- | --- | --- | --- |
| $k$ | unitless | Gradient of fibre decay rate | 0.024 | 0.0258 | 7.98$\times{10}^{-4}$ | **Fig 3**B |
| $D_{const}$ | 1/hour | Constant fibre decay rate | $8.14\times{10}^{-4}$ | $5.1\times{10}^{-13}$ | $9.33\times{10}^{-4}$ | **Fig 3**B |
| $C_{0}$ | $\mu g$ | Initial drug concentration | 6.95 | 35.78 | 483.8431 | **Fig 3**B |
| $A_{out}$ | $\mu m^{2}$ | Area outside the fibre | $382.3$ | 1408 | 5989 | **Fig 3**B |
| $\epsilon$ | hour | Singularity perturbation | 1 | 1 | 1 | Estimated |
| $L$ | $\mathrm{cm}$ | Length of fibre | 0.5 | 0.5 | 0.5 | [1, 2] |
| $r_{total}$ | $\mu m$ | Radius of the fibre | 19 | 38 | 688 | [1, 2] |

**Table B. Gemcitabine decay and induced cell death kinetics.** Parameter values for gemcitabine drug effect, obtained from fitting **Eqs. (6)-(8)** to cell viability measurements (**Fig 3**F) for Mia-PaCa-2 cells. To fit the cell induced death kinetic parameters $\delta$ and $IC_{50}$ we needed to also fit the proliferation rate of cells in vitro (**Fig 3**D). This in vitro proliferation rate $r$ was not re-used in the full VCBM-PDE model as we were approximating in vivo tumour growth which differs significantly from in vitro tumour cell proliferation.

| **Params Units** | | **Description** | Value | Ref |
| --- | --- | --- | --- | --- |
| $\delta_{m}$ | $1$/hour | Drug-induced cell death rate | 0.067 | **Fig 3**F |
| $IC_{50}$ | $\mu g$ | Half-conc for maximal drug-induced Mia-PaCa-2 cell death | 10 | **Fig 3**F |
| $\lambda$ | 1/hour | Decay rate of drug | 0.0357 | [3] |
| $\phi$ | 1/hour | *In vitro* proliferation rate of Mia-PaCa-2 cells. | 0.0132 | **Fig 3**D |

**Table C. Agent-related parameters values for the VCBM.** To estimate cell proliferation parameters, we did a simple Latin Hypercube Sampling and found a parameter set that minimised the least squares distance between the control in vivo Mia-PaCa-2 tumour volume and the model prediction (**Fig 4**B). Other parameters, particularly related to cell motility, were fixed to their value estimated in our previous VCBM development [4].

| **Param** | **Units** | **Description** | Value | Ref |
| --- | --- | --- | --- | --- |
| Cell proliferation parameters | | | | |
| $p_{0}$ | - | Proliferation probability constant | 0.2 | LHS |
| $d_{max}$ | mm | Maximum radial distance for nutrients | 1.7 | LHS |
| $g_{age}$ | hours | Time for daughter cell to grow to adult cell size | 170 | LHS |
| $p_{age}$ | Hours | Time from mitosis to next cell cycle division | 2 | [4] |
| $p_{MCC}$ | - | Probability of an MCC being created in $\Delta t_{cells}$ | ${10}^{-4}$ | LHS |
| $\Delta t_{cells}$ | Hours | Time step for cell action | 1 | [4] |
| $r_{min}$ | $\mu m$ | Minimum distance between neighbouring cells for proliferation | 3 | [4] |
| Cell motility parameters | | | | |
| $s$ | $\mu m$ | Spring rest length | 18.5 | [4] |
| $\lambda_{m}$ | hour | Cell mobility constant |  | [4] |
| $a_{l}$ | $\mu m$ | Adhesion distance between two cells | 0.15 | [4] |

**Table D. Remaining parameters for drug diffusion in the tumour microenvironment (TME) and the Finite Volume Method (FVM) approximation.**

| Params Units | | Description | Value |
| --- | --- | --- | --- |
| $\Delta$ | $\mu m$ | Spatial discretization ($\Delta x=\Delta y=\Delta)$ | 1 |
| $l$ | $\mu m$ | Length of the domain (**Fig A in S1 Technical Supplementary Information**) | $100$ (initially) |
| $w$ | $\mu m$ | Width of the domain (**Fig A in S1 Technical Supplementary Information**) | $100$ (initially) |
| $d_{age}$ | hours | Time taken for a dead cell to disintegrate | 3 |
| $\sigma$ | 1/mm | Scaling from $\mu m$ to $mm$ | 0.1728 |
| $\nu_{C}$ | 1/min | Uptake of drug by cells | 0.01 |
| $D$ | $\mu m/min$ | Diffusion coefficient | 0.01 |
| $h$ | $\mu m$ | Height of the 2D cross section (**Fig A in S1 Technical Supplementary Information**) | 1 |

**Table E. Full list of model parameters and variables in the VCBM-PDE main text listed in alphabetical order**

| Symbol | Unit | Description |
| --- | --- | --- |
| $A_{out}$ | $\mu m^{2}$ | Area outside the fibre |
| $a_{l}$ | $\mu m$ | Adhesion distance between two cells |
| $B$ | Dimensionless | Boundary of 2D rectangular domain considered for the model |
| $C$ | $\mu g/\mu m$ | Concentration of drug in the tumour microenvironment |
| $C_{0}$ | $\mu g$ | Initial drug concentration |
| $F$ | $\mu g/\mu m$ | Drug concentration inside the fibre |
| $D$ | $\mu m^{2}/min$ | Diffusion coefficient for the drug gemcitabine |
| $D_{F}$ | $\mu m^{2}/min$ | Time-dependent diffusion of drug inside fibre |
| $d_{age}$ | Hour | Time taken for a dead cell to disintegrate |
| $D_{const}$ | 1/hour | Constant release rate |
| $d_{neut}$ | $\mu m$ | Distance of cell from nearest edge of the tumour |
| $d_{max}$ | $\mu m$ | Maximum nutrient distance |
| $d$ | 1/hour | Drug release rate from alginate fibre |
| $d_{m}$ | $\mathrm{mm}$ | Distance from the centre of the tumour |
| $\delta_{m}$ | 1/day | maximum death rate due to gemcitabine |
| $\Delta$ | $\mu m$ | Spatial discretization ($\Delta x=\Delta y=\Delta$) |
| $\Delta t$ | Hour | Time step |
| $\epsilon$ | min | Constant to remove the singularity in the diffusivity **Eq. (2)** |
| $\eta$ | $\mu g.hour$ | Half-effect of the drug on the Emax, Imax release curves |
| $\gamma$ | 1/min | Rate constant in release profiles considered |
| $g_{age}$ | Hour | Age cell needs to reach before it can proliferate |
| $h$ | $\mu m$ | Height of the 2D rectangular tumour cross section (**Fig A in S1 Technical Supplementary Information**) |
| $IC_{50}$ | $\mu g$ | Half-effect of the drug on PDAC cell apoptosis |
| $K$ | Dimensionless | Total intervals considered in x-direction in domain $B$for FVM |
| $k$ | 1/min | Gradient of release rate from fibre |
| $\lambda$ | 1/min | Decay rate of gemcitabine |
| $\lambda_{m}$ | hour | Cell mobility constant |
| $L$ | $\mu m$ | Length of fibre |
| $l$ | $\mu m$ | Length of domain |
| $M$ | Dimensionless | Number of annuli |
| $N$ | Dimensionless | Total intervals considered in y-direction in domain $B$for FVM |
| $N_{inj}$ | Dimensionless | Number of infections |
| $\vec{n}$ | Dimensionless | Outward unit normal on the boundary $B$ |
| $P_{L}$ | Cells | Live cancer cells |
| $P_{D}$ | Cells | Dead cancer cells |
| $p_{age}$ | Hour | Time between division and next possible division |
| $p_{d}$ | Dimensionless | Probability of a cell dividing |
| $p_{0}$ | Dimensionless | Proliferation constant |
| $p_{MCC}$ | Dimensionless | Probability of a cell differentiating into a MCC and PDAC cell. |
| $\phi$ | 1/hour | Tumour cell proliferation rate |
| $r$ | $\mu m$ | Radial position of gemcitabine inside the fibre |
| $r_{min}$ | $\mu m$ | Minimum distance needed between cells before they can proliferate |
| $r_{total}$ | $\mu m$ | Total length of the fibre |
| $\vec{r}_{k}$ | $\mu m$ | Vector for the position of cell $k$ |
| $s$ | $\mu m$ | Spring rest length |
| $s_{k,i}$ | $\mu m$ | Spring rest length between cell $k$ and $i$ |
| $\sigma$ | $1/mm$ | Scaling the unit length in the model to a $\mathrm{mm}$ length |
| $t$ | Hour | Time |
| $t_{aliquot}$ | Hours | Times of drug administrations |
| $\theta$ | rad | Rotation of newly divided daughter cells |
| $v_{c}$ | 1/min | Gemcitabine uptake rate by cells |
| $V$ | $\mu m$ | Volume of voxel |
| $W_{k}$ | $\mu m^{3}$ | Volume of cancer cell |
| $w$ | $\mu m$ | Width of domain |
| $x_{F}$ | Variable | Location of line source representing fibre in FVM discretization |
| $x_{inj}$ | Variable | x-position where drug injection takes place |
| $y_{inj}$ | Variable | y-position where drug injection takes place |
| $y_{0}$ | Variable | $y$-position of the bottom of the fibre in the FVM discretization |


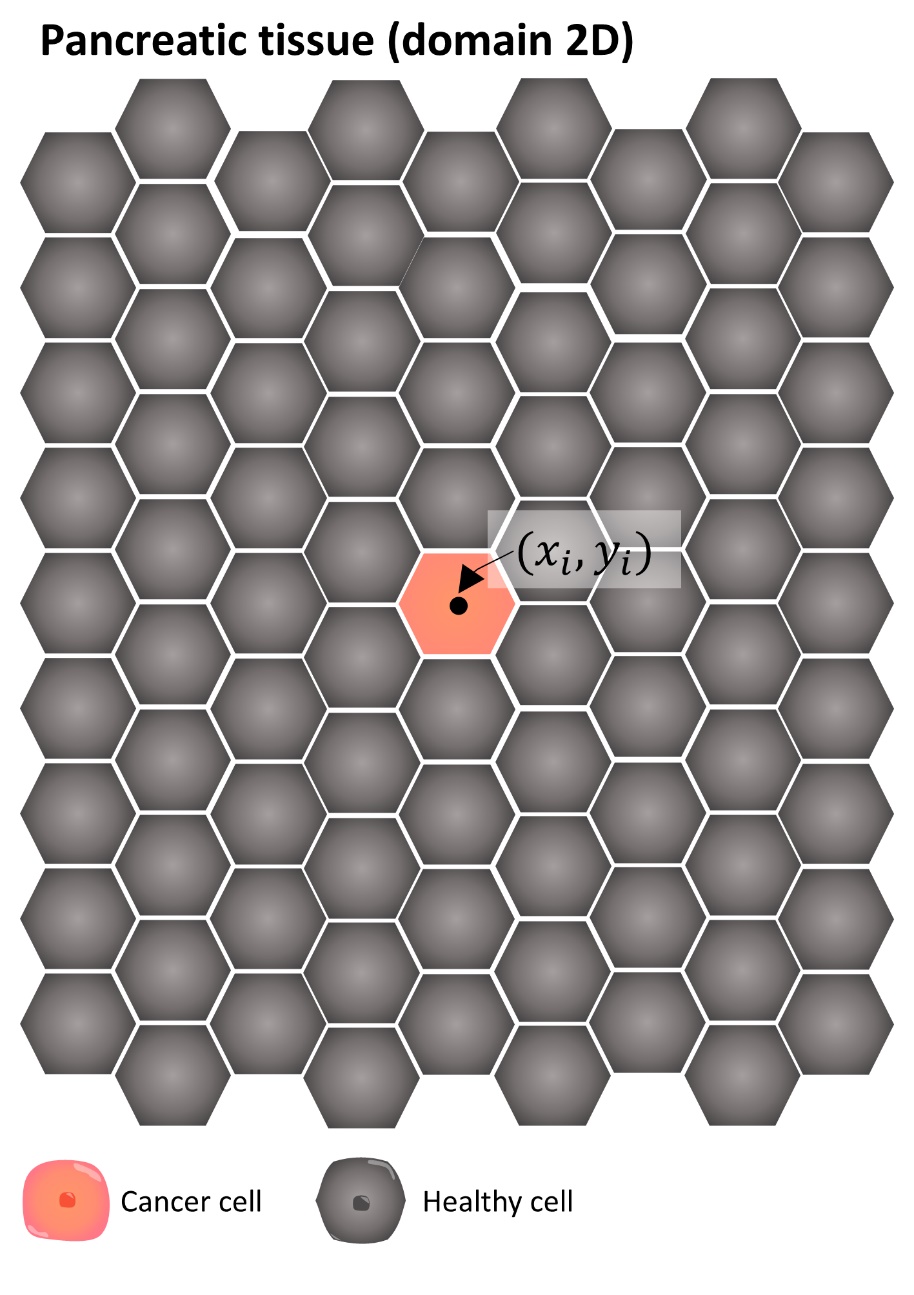


**Fig A. Initialisation of the simulation.** The VCBM model is first initialised by placing a single cancer cell randomly in the centre of the domain, where cells initially are tessellated in a hexagonal arrangement and the remainder of cells are healthy pancreatic tissue cells. The model is then simulated until the tumour reaches a tumour volume that matches the $in vivo$ initial tumour volume. The centre of the cell is represented by the point $(x_{i},y_{i})$. Legend for cell colouring: cancer cell (orange) healthy cell (grey).

**
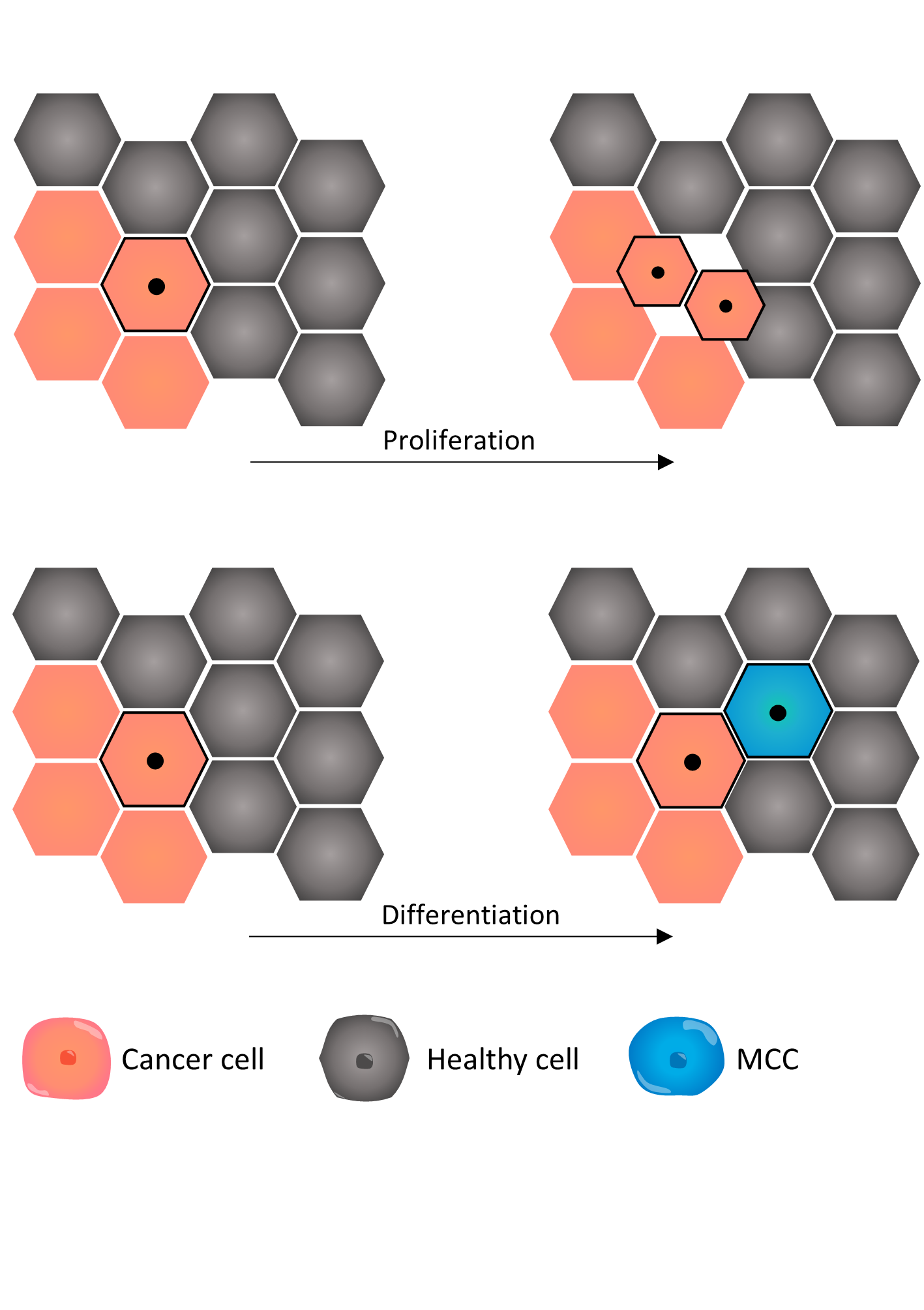
**

**Fig B. Tumour cell proliferation and differentiation.** In the VCBM, tumour cells can either undergo proliferation or differentiation. We assume when a tumour cell proliferates it creates two new daughter cells that are both considered tumour cells. In comparison, when a tumour cell differentiates, we assume it creates a new MCC which is placed on a neighbouring healthy cell, and that healthy cell is removed from the model.

**
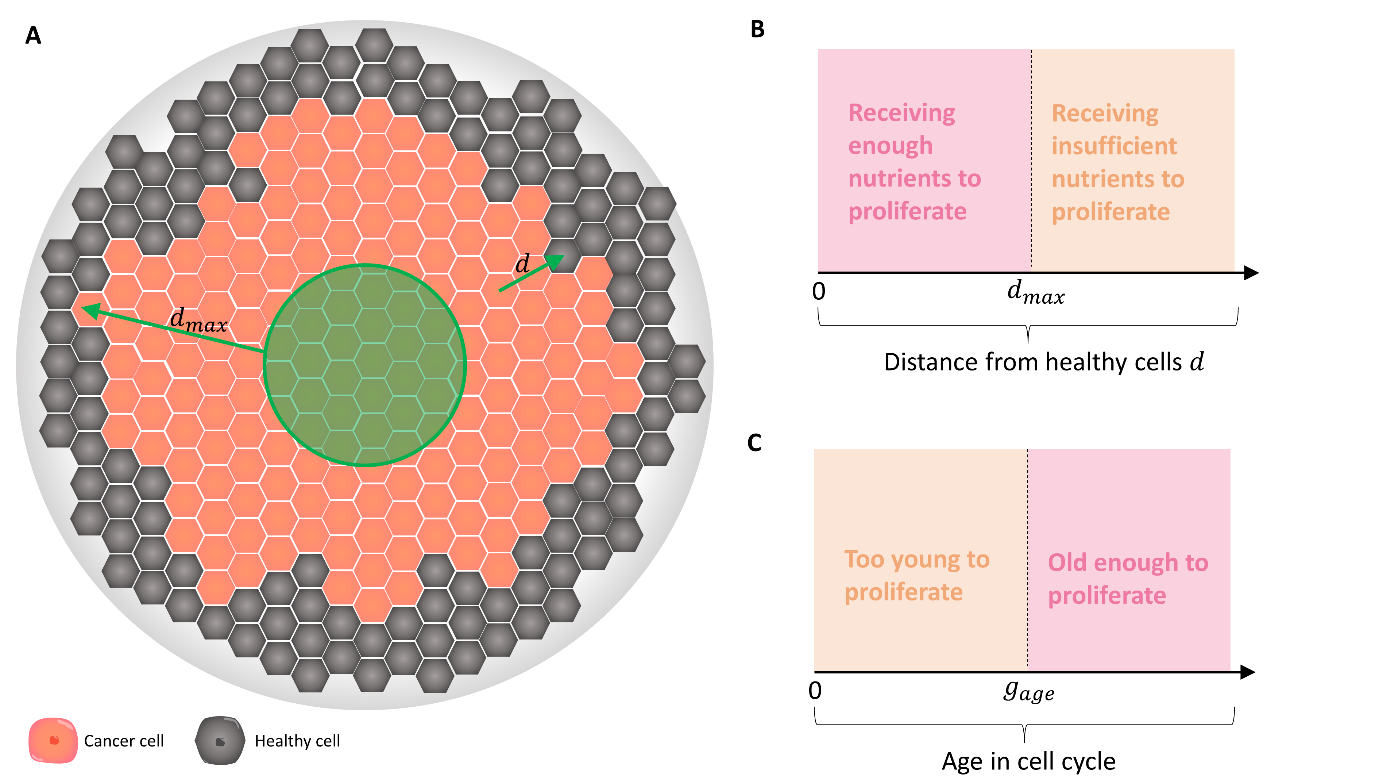
**

**Fig C. Schematic for the probability of a cell proliferation given its distance from the edge of the tumour and age in the cell cycle.** (A) For a given cell, the distance from the edge of the tumour is denoted by $d$. The farthest distance for proliferation is given by $d_{max}$. (B) Cells will proliferate if they are receiving enough nutrients to proliferate, i.e. $d<d_{max}$, and will not proliferate otherwise. (C) Cells will proliferate if their age in the cell cycle is greater than $g_{age}$. Legend for cell colouring: cancer cell (orange) healthy cell (grey).


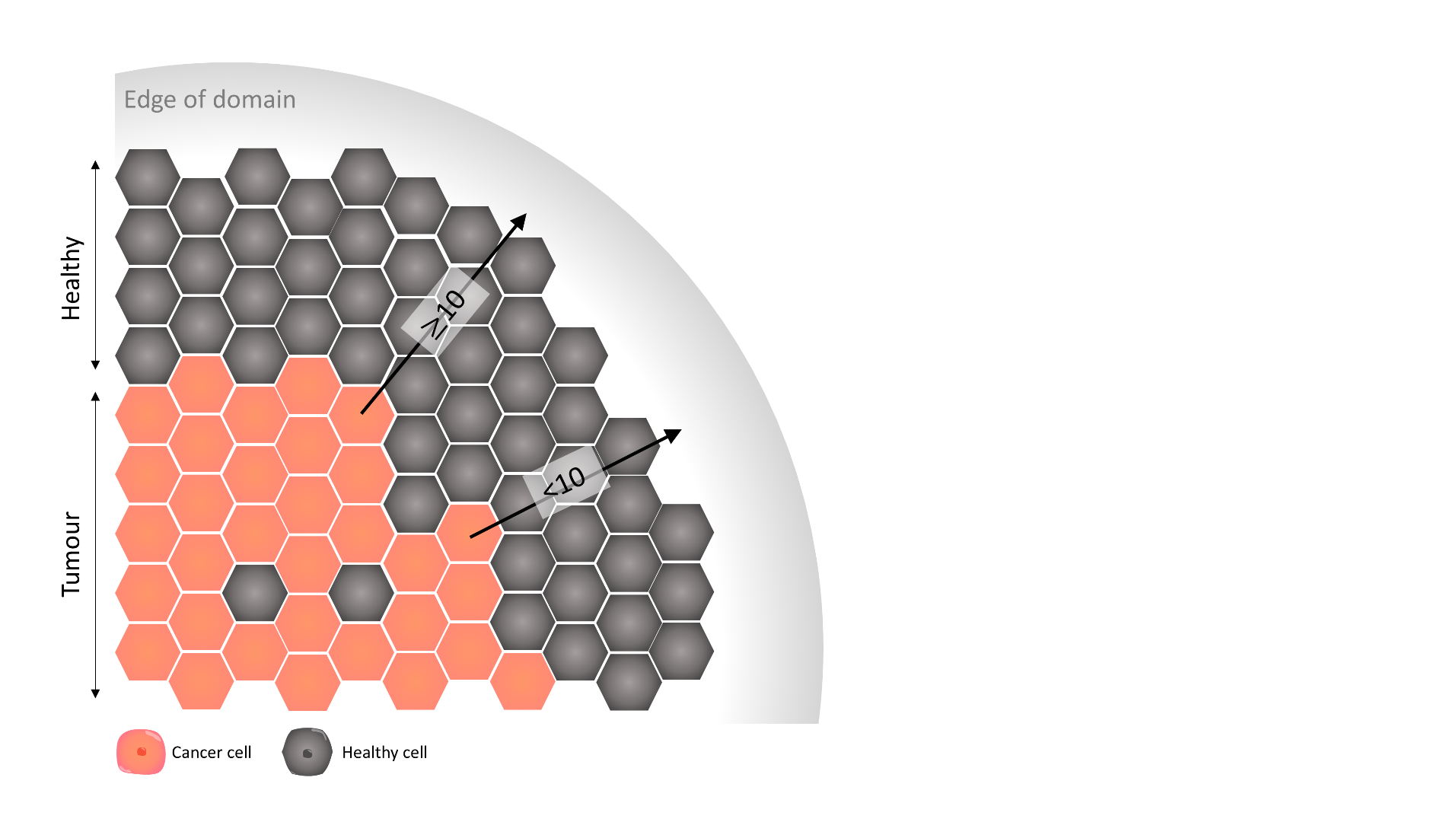


**Fig D. Expanding the simulation domain.** As the tumour expands, we need to increase the simulation domain so that no tumour cells are left with empty space as a neighbour. To do this, we check whether any tumour cell is within $10$ units of the edge of the domain (or empty space of our simulation). If any satisfy this condition, new healthy cells are adding by expanding the lattice to include cells in an annulus of radius $10$ units at the edge of the domain. Legend for cell colouring: cancer cell (orange) healthy cell (grey).


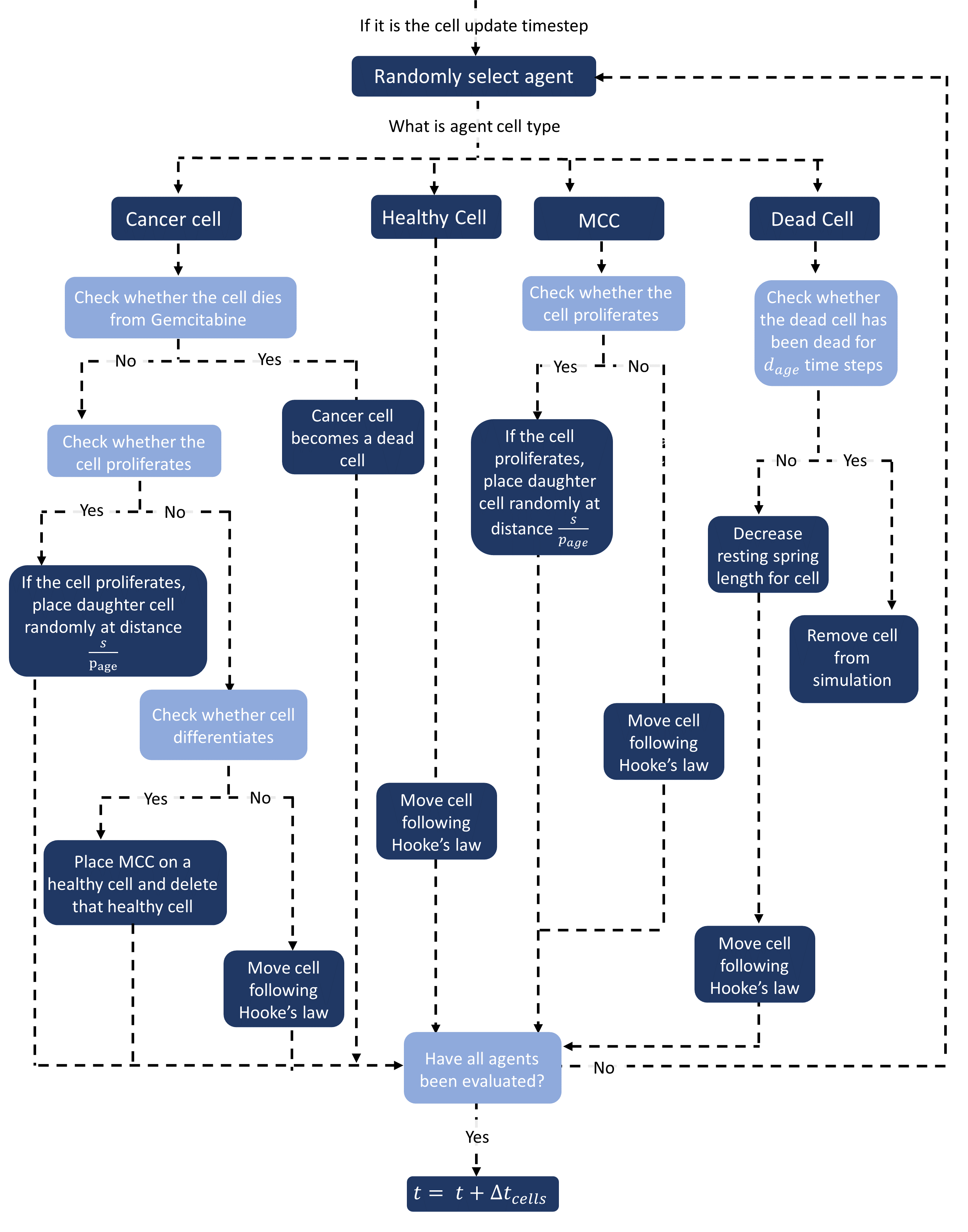


**Fig E. Decision tree diagram detailing the modelling rules used for updating cells in the VCBM.** When the model has reached the cell update time-step $\Delta t_{cells}$ (see **Fig C in S1 Technical Supplementary Information**), the cells are updated. To update cells the process in this schematic is followed. This process is detailed by the assumptions and rules provided in the main text.


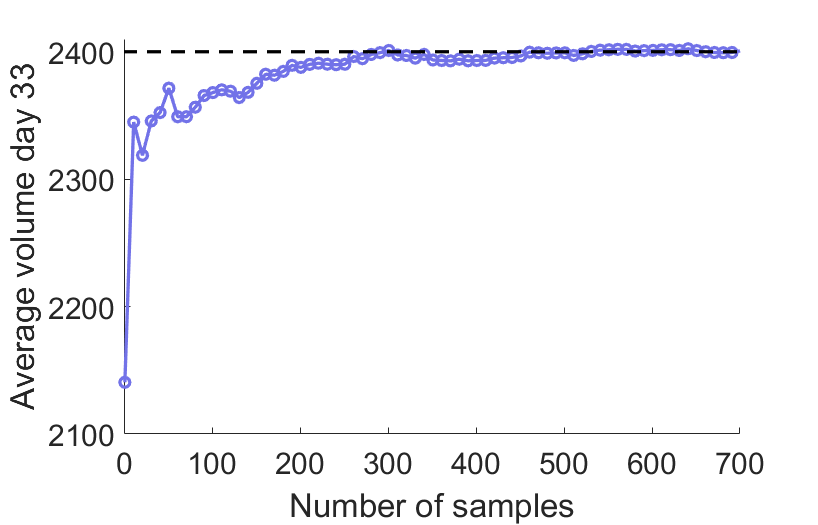


**Fig F. Convergence of stochasticity in the VCBM.** To capture the level of stochasticity in the VCBM we have plotted the average tumour volume on day 33 in the untreated case, where the average is plotted for the corresponding number of simulation samples on the horizontal axis. For example, 100 samples corresponds to 100 realisations of the model. From this, we can see that the model achieves convergence of the mean by 500 samples, and we now fix the number of samples that we need to simulate the model as $n=500$.


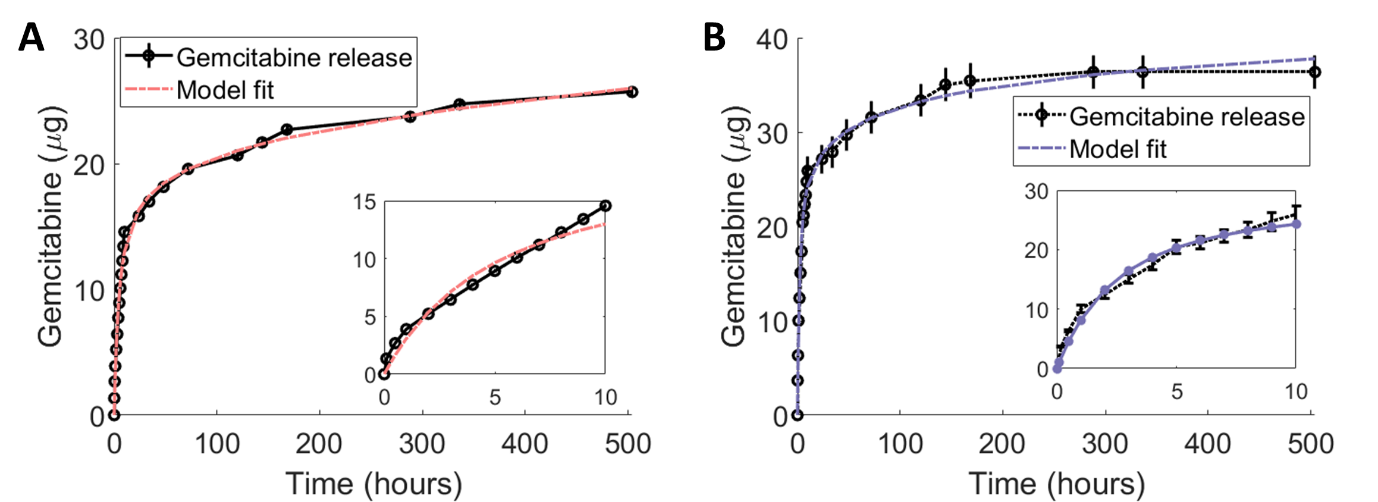


**Fig G. Optimisation of fibre release kinetics.** Release profiles were measured from fibres created from different concentrations of alginate. Cumulative release of gemcitabine was measured from 1% alginate (A), 2% alginate (B) and 3% alginate (**Fig 3**B). Values are given for mean ($\pm$SEM) of triplicates (black). Overlaid is the fitted model simulation (dash dot coloured line). The parameter values obtained for these curves are in **Table A**. Parameters for all model simulations were taken from the 3% alginate fit, see **Fig** 3B, and the fits plotted here for 1% and 2% alginate are provided only as a validation of the model’s ability to capture varying fibre release profiles.


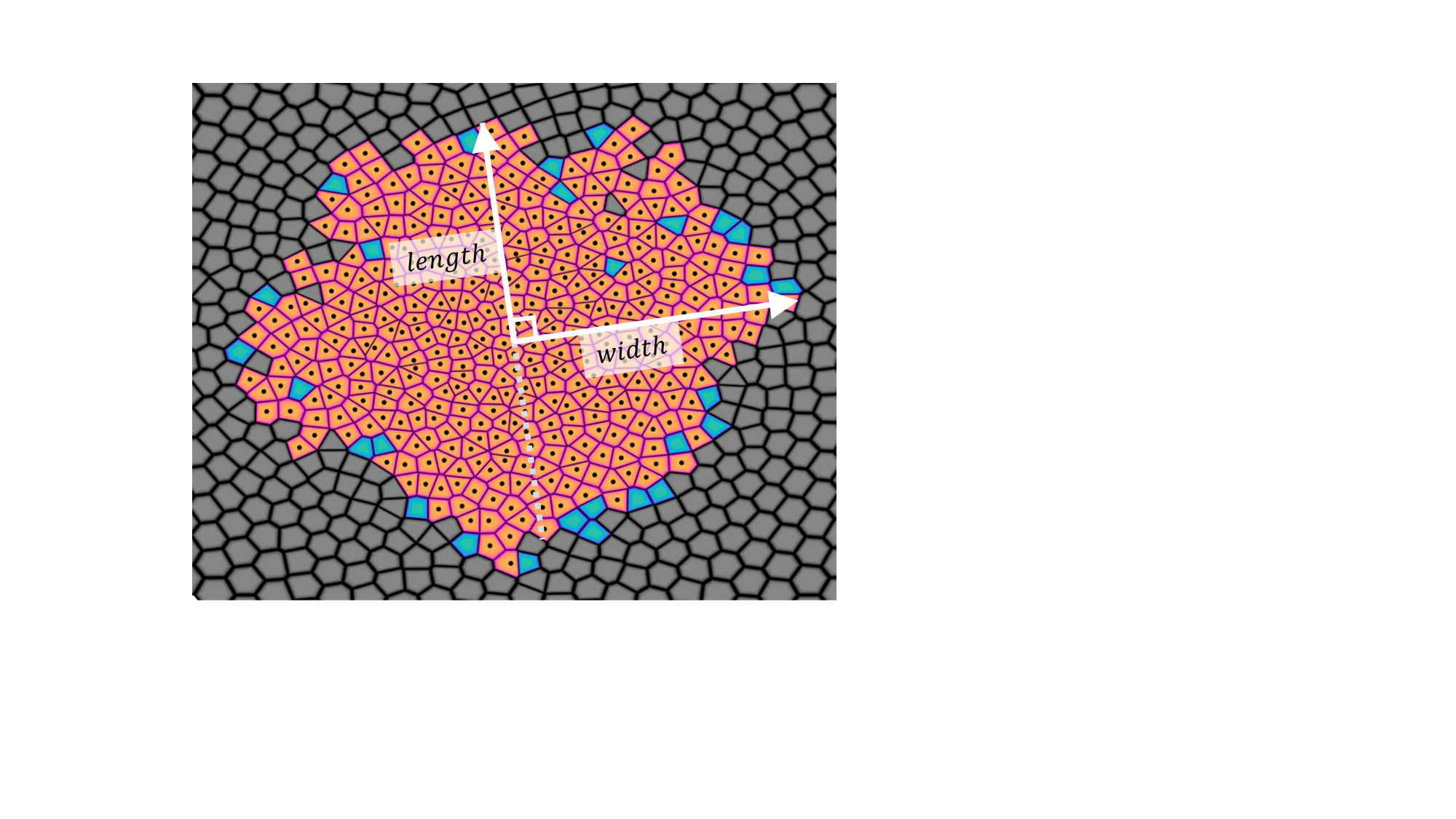


**Fig H. Depiction of the tumour volume approximation calculation.** The longest radial axis was determined by finding the cell centre farthest from the centre of the tumour. The distance between this cell and the tumour centre is denoted by $w$. The farthest cell along the perpendicular radial axis was then used to determine the length $l$ which is the distance from that cell to the tumour centre. These measurements were then used to determine an approximate tumour volume. Legend for cell colouring: cancer cell (orange) healthy cell (grey), MCC (blue).


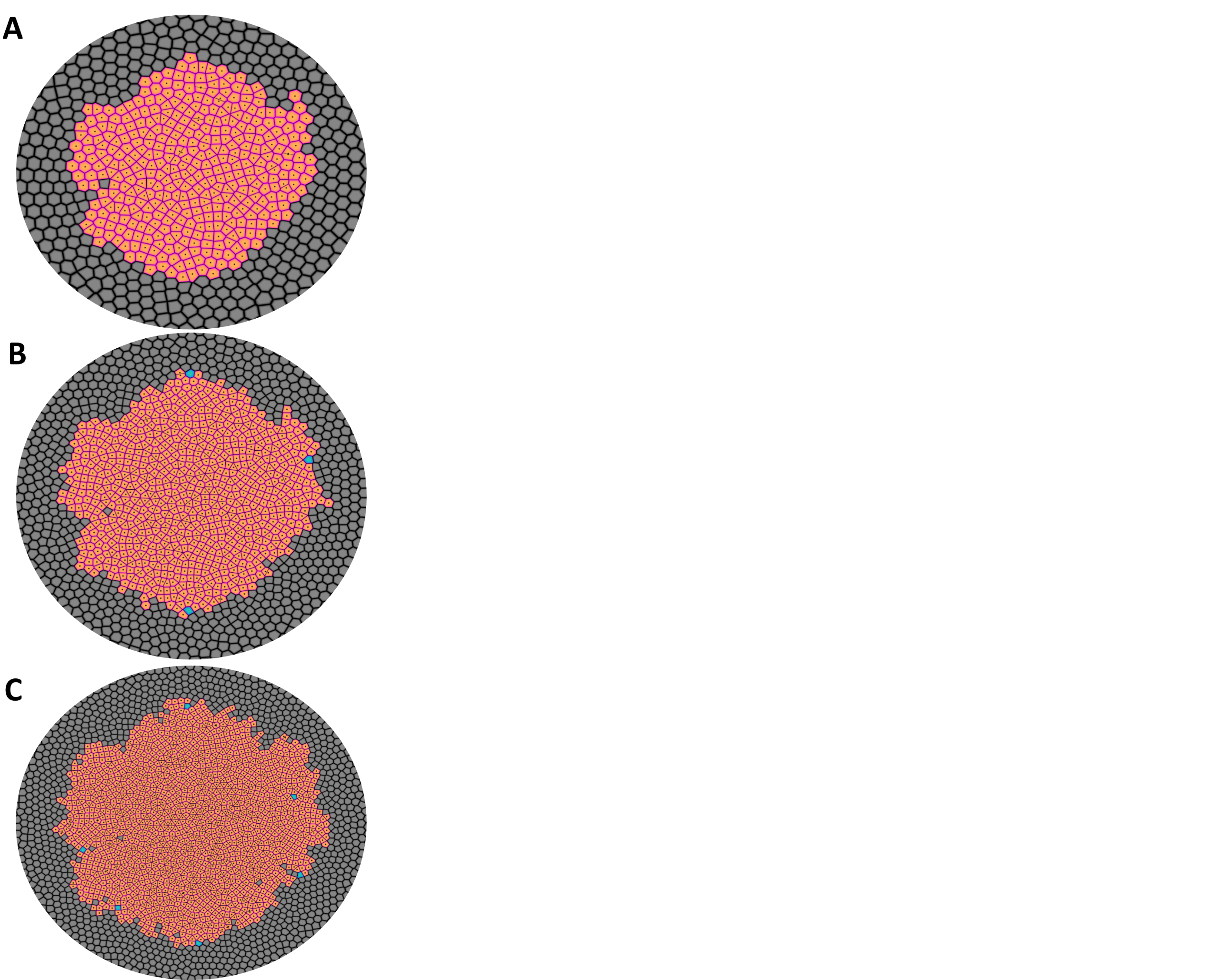


Cancer cell

MCC

Healthy cell

**Fig I. Larger reproduction of the figures in Fig 4A**. The figures in **Fig 4A** have been reproduced here in a larger format so that readers can inspect them in more detail. Legend for cell colouring: cancer cell (orange) healthy cell (grey), MCC (blue).


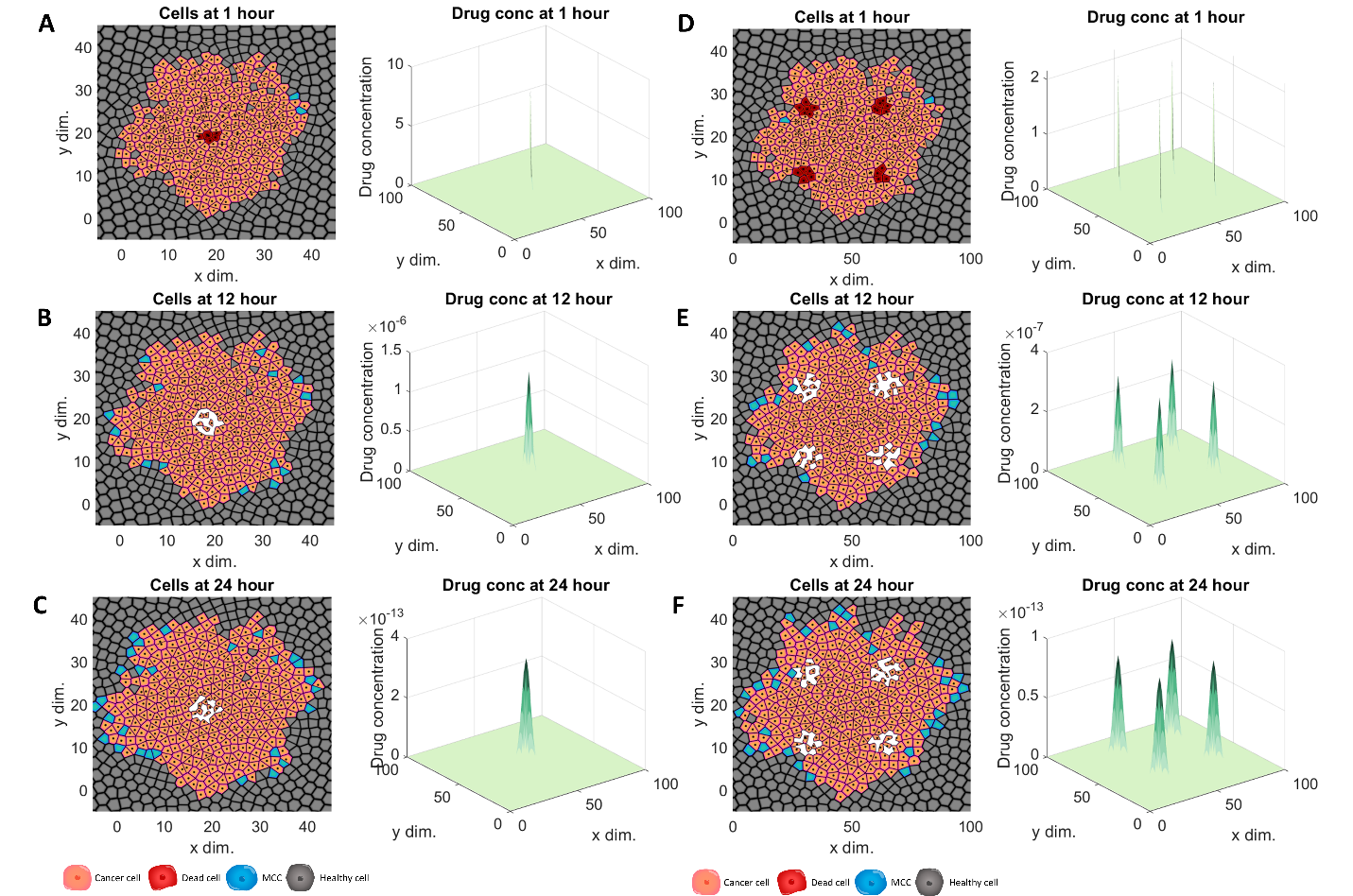


**Fig J. Evolution of tumour volume under injection with gemcitabine.** (A)-(B) VCBM-PDE model under a single centered injection of gemcitabine. (D)-(F) VCBM-PDE model under four injections of gemcitabine. Plots are given for simulation at (A)&(D) 1hr, (B)&(E) 12 hours and (C)&(F) 24 hours. The PDE concentration of gemcitabine is pictured as a surface plot and the initial cell agents are coloured according to the legend. Note the unit dimensions are those of the simulation and have not been converted to $mm$ or $\mu m$. Legend for cell colouring: cancer cell (orange) healthy cell (grey), MCC (blue), dead cell (red). In these simulations, we present the $x,y$ values in their model form, but we translate these in the main text for the comparison to the data $\sigma$.


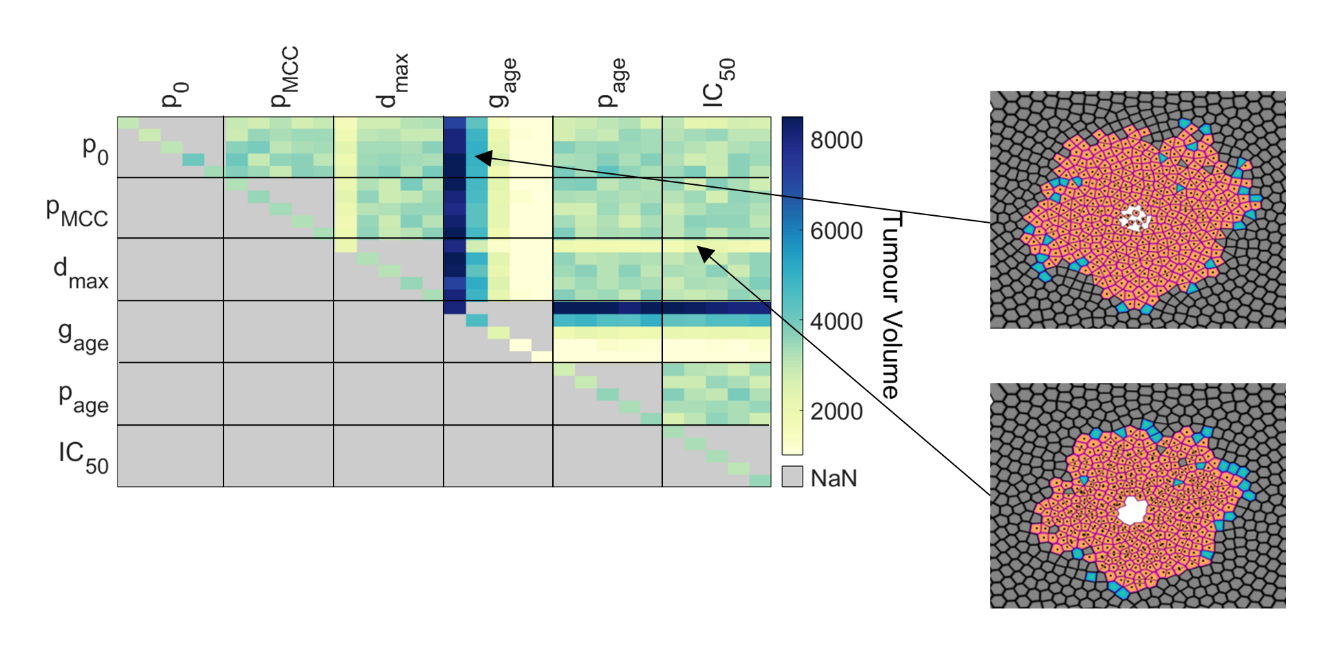


**Fig K. Multi-parameter sensitivity for single injection.** Systematic multi-parameter analysis for a single injection model over 33 days produced in a similar manner to **Fig 4**D. Each parameter has been weighted 0.25, 0.75, 1.25, 1.75 and 2.25 in each box, with each pixel representing two parameters holding one of these weightings. For each pixel, 10 simulations were run over 33 days and the results averaged, before the maximum volume was found. The same trends for $g_{age}$ and $d_{max}$ found in the control model still hold. The different tumours for the indicated weightings can be shown at 24 hours on the right. Legend for cell colouring: cancer cell (orange) healthy cell (grey), MCC (blue), dead cell (red). Note, unlike the simulations in the main text where 500 simulations were used for all result figures, this figure was created as the average of 30 simulations of each parameter set.


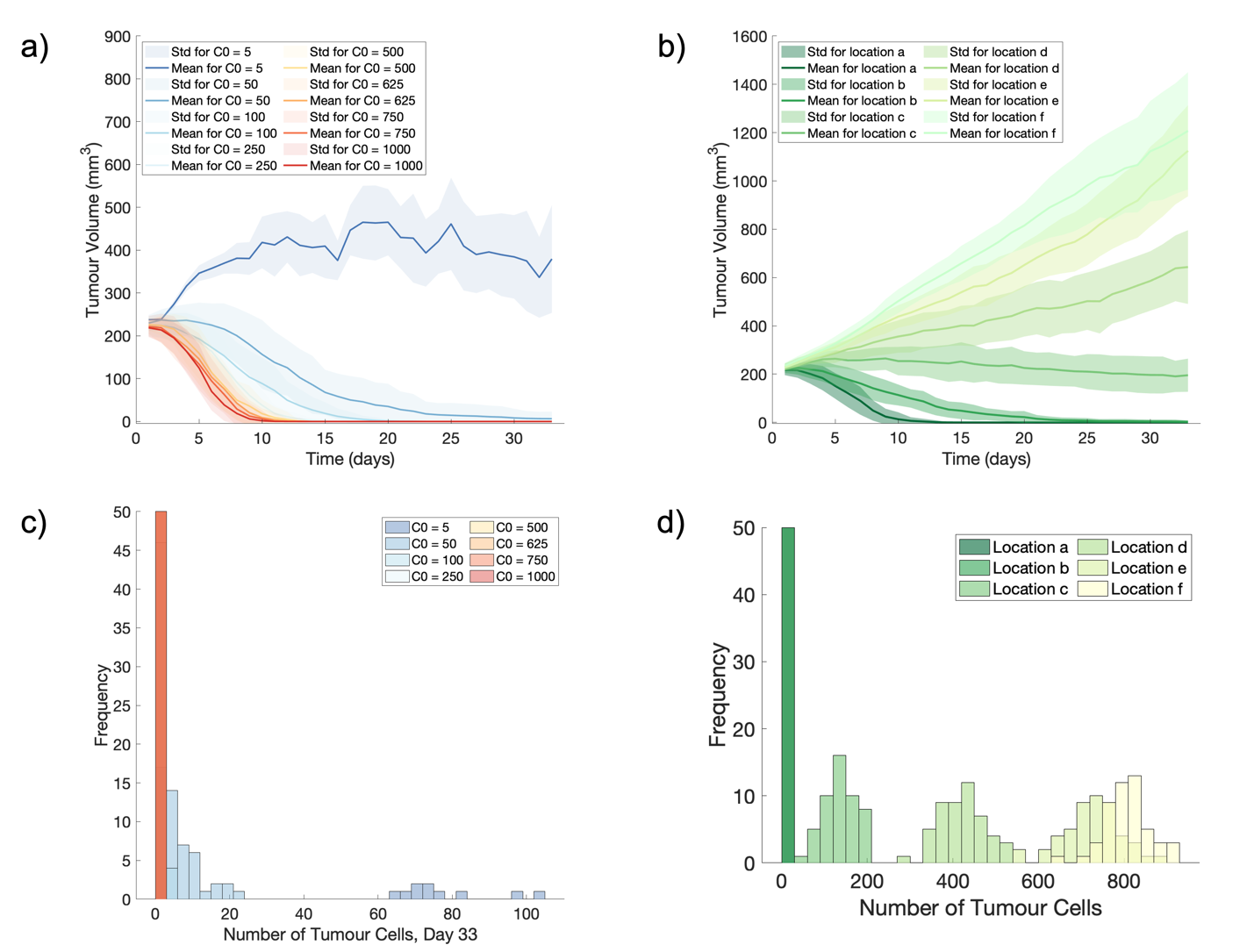


**Fig L. Effect initial injection concentration and fibre placement have on tumour size on day 33 (the final day of simulation).** (A) Mean (solid lines) and standard deviation (shaded areas) Of tumour volume over 33 days with varying initial injection concentrations. (B) Means (solid lines) and standard deviation (shaded areas) of tumour volume over 33 days with varying fibre location. Here location a is central, b is 0.9 mm from the centre, c is 1.7 mm from the centre, d is 2.5 mm from the centre, e is 3.5 mm from the centre and f is 4.3 mm from the centre. (C) Histogram of the final number of tumour cells present in the simulation area for varying initial injection concentrations. (D) Histogram of the final number of tumour cells present in the simulation area for varying fibre placements. Note, unlike the simulations in the main text where 500 simulations were used for all result figures, this figure was created as the average of 30 simulations of each parameter set.


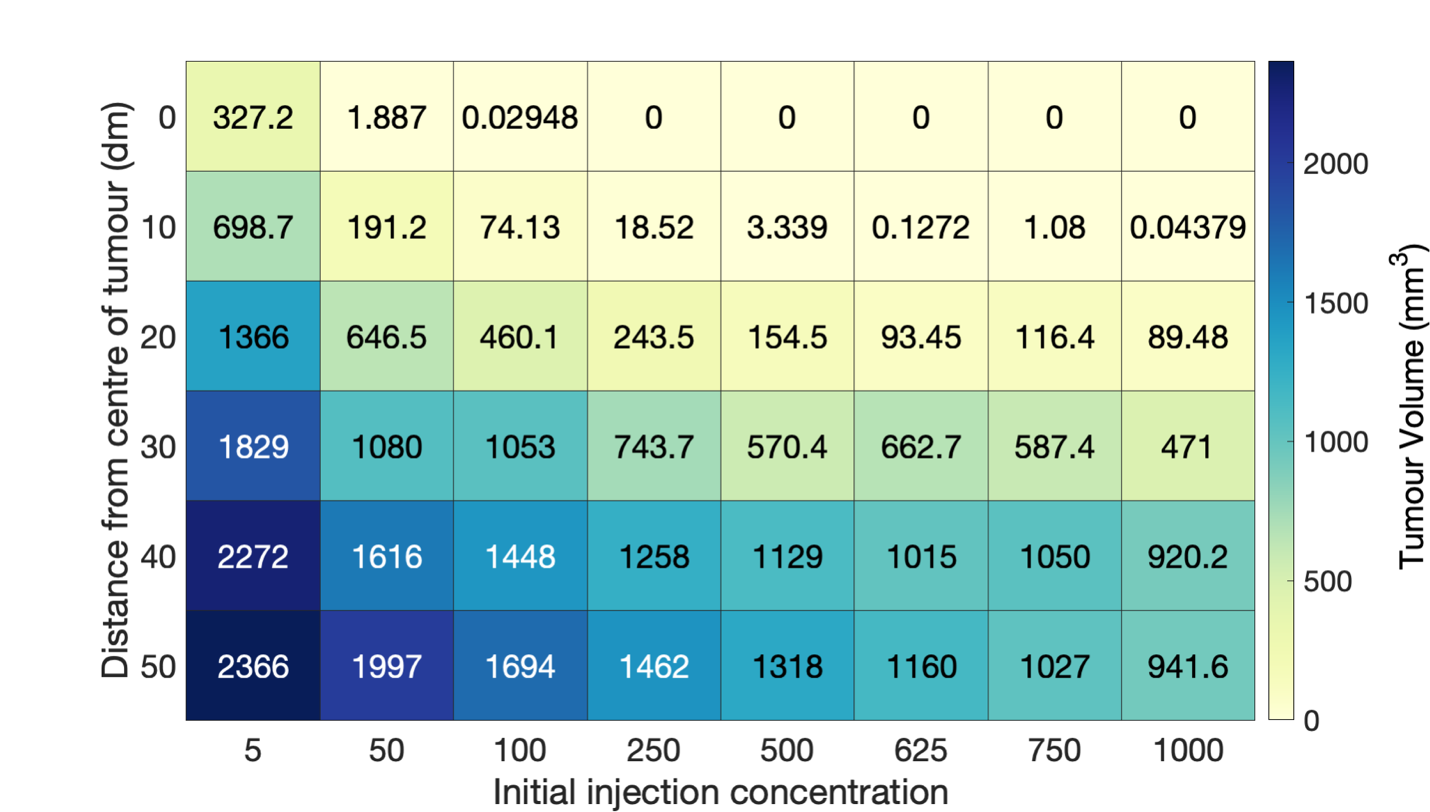


**Fig M.** Average tumour volume on day 33 for varying initial injection concentrations and fibre placements. Note, unlike the simulations in the main text where 500 simulations were used for all result figures, this figure was created as the average of 30 simulations of each parameter set. Note, the units denoted by distance in this heatmap are not in dimensionless form (i.e. $mm)$


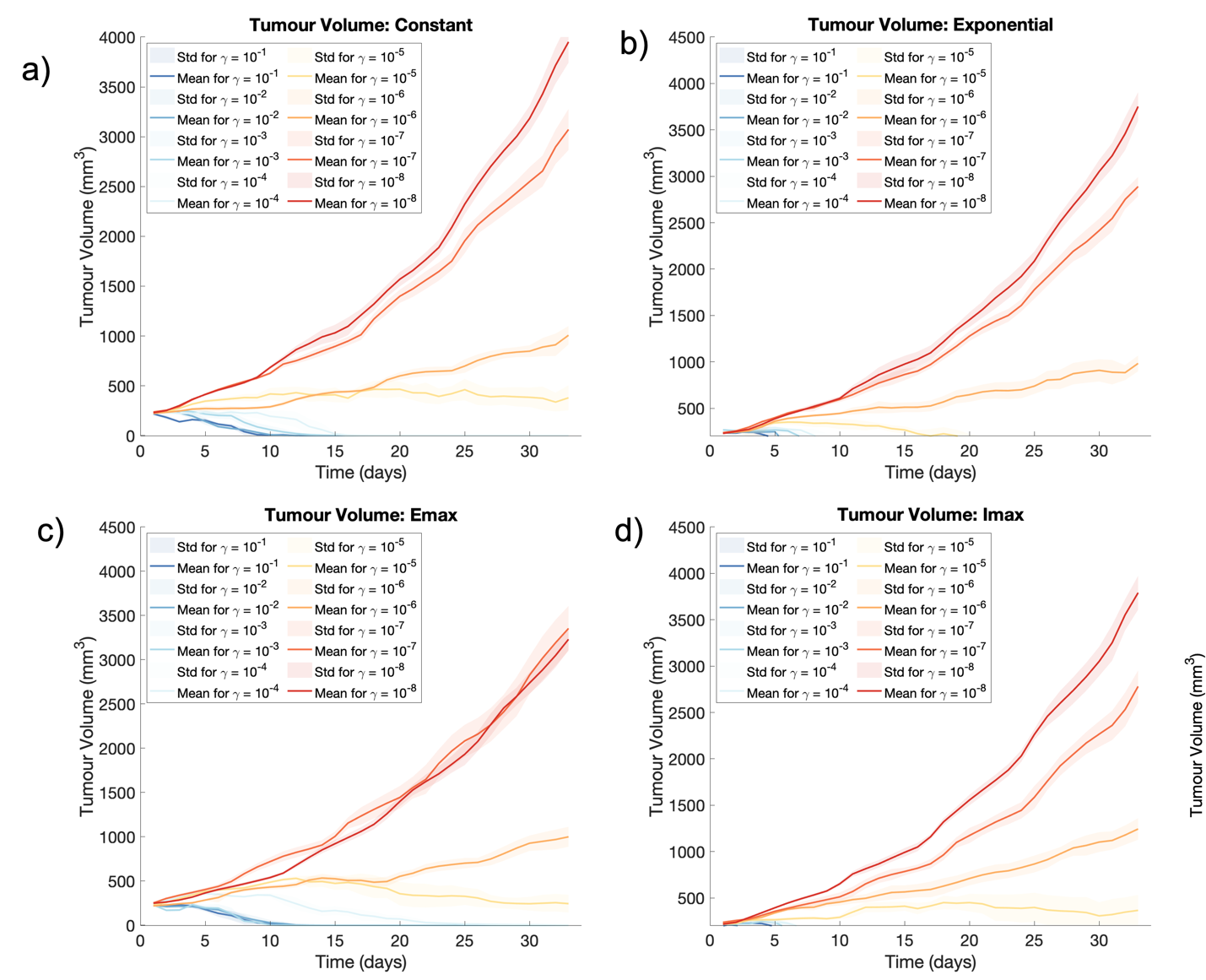


**Fig N. Tumour volume for different release profiles.** (A) Tumour volume over time for a constant release rate, with varying values of gamma. (B) Tumour volume over time for an exponential release rate, with varying values of gamma. (C) Tumour volume over time for a sigmoidal Emax release rate, with varying values of gamma. (D) Tumour volume over time for a sigmoidal Imax release rate, with varying values of gamma. See **Section TS3 in S1 Technical Supplementary Information** for more information on these release functions. Note, unlike the simulations in the main text where 500 simulations were used for all result figures, this figure was created as the average of 30 simulations of each parameter set.


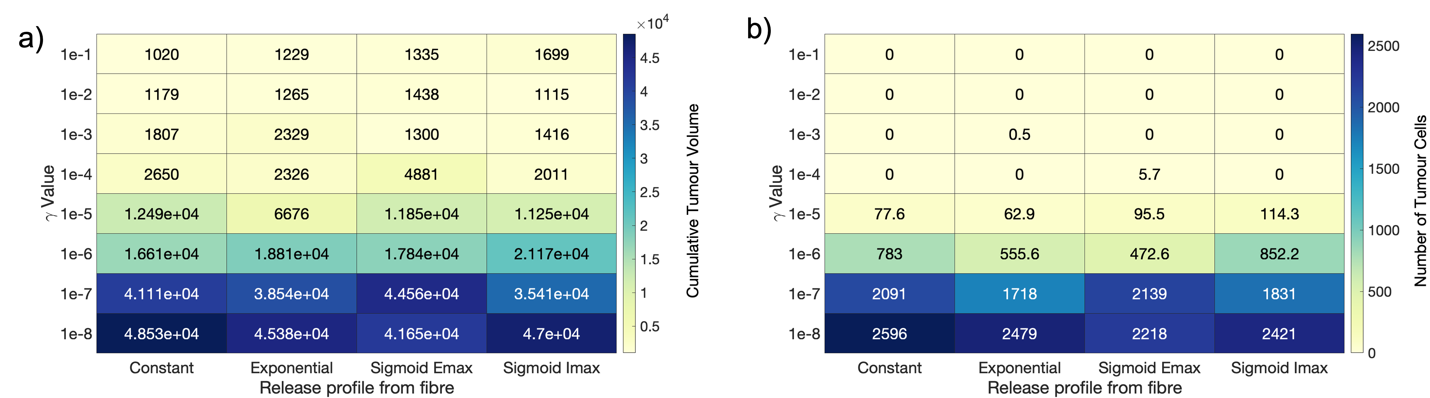


**Fig O.** Figures for different release profiles from fibre, for a central placement. (A) Cumulative tumour volume over time for varying gamma values and release profiles. (B) Number of tumour cells over time for varying gamma values and release profiles. Note, unlike the simulations in the main text where 500 simulations were used for all result figures, this figure was created as the average of 30 simulations of each parameter set.

**References**

1. Wade SJ, Zuzic A, Foroughi J, Talebian S, Aghmesheh M, Moulton SE, et al (2017) Preparation and in vitro assessment of wet-spun gemcitabine-loaded polymeric fibers: Towards localized drug delivery for the treatment of pancreatic cancer. Pancreatology 17:795–804

2. Wade SJ, Sahin Z, Piper A-K, Talebian S, Aghmesheh M, Foroughi J, et al (2020) Dual Delivery of Gemcitabine and Paclitaxel by Wet-Spun Coaxial Fibers Induces Pancreatic Ductal Adenocarcinoma Cell Death, Reduces Tumor Volume, and Sensitizes Cells to Radiation. Adv Healthc Mater 9:2001115

3. Kinikar SA, Kolesar JM (1999) Identification of a Gemcitabine-Warfarin Interaction. Pharmacother J Hum Pharmacol Drug Ther 19:1331–1333

4. Jenner AL, Frascoli F, Coster ACF, Kim PS (2020) Enhancing oncolytic virotherapy: Observations from a Voronoi Cell-Based model. J Theor Biol. https://doi.org/10.1016/j.jtbi.2019.110052
