# Supplementary material for: Examining the efficacy of localised gemcitabine therapy for the treatment of pancreatic cancer using a hybrid agent-based model
Source: PLoS Comput Biol. 2023 Jan 17;19(1):e1010104. doi: 10.1371/journal.pcbi.1010104 (PMC9891514; doi:10.1371/journal.pcbi.1010104)
Supplement: S1 Technical Supplementary Information — Includes Figs A-F. (DOCX) [file pcbi.1010104.s002.docx]

**Technical Supplementary Information: Examining the efficacy of drug-loaded polymers in the treatment of pancreatic cancer using a hybrid agent-based model.**

Adrianne L. Jenner^1^, Wayne Kelly^2^, Michael Dallaston^1^, Robyn Araujo^1^, Isobelle Parfitt^1^, Dominic Steinitz^3,4^, Pantea Pooladvand^5^, Peter S. Kim^5^, Samantha J Wade^6^, Kara L. Vine^6,7^,

**Contents**

[TS1 Extended description of the VCBM-PDE model 2](#_Toc98839192)

[TS2 Numerical approximation for gemcitabine concentration in the tumour microenvironment 4](#_Toc98839193)

[TS2.1 Drug diffusion in 2-dimensions in the TME 4](#_Toc98839194)

[TS2.2 Outer TME boundary conditions 5](#_Toc98839195)

[TS2.3 Drug diffusion within the fibre 6](#_Toc98839196)

[TS2.4 Cell uptake and time discretization 7](#_Toc98839197)

[TS3. Fibre release functions 7](#_Toc98839198)

[TS4 References 8](#_Toc98839199)

# TS1 Extended description of the VCBM-PDE model

In this document, we summarize the hybrid Voronoi Cell-Based model (VCBM)-partial differential equation (PDE) model for the treatment of pancreatic ductal adenocarcinoma cancer (PDAC) tumours with the drug gemcitabine. To model the concentration of the drug gemcitabine in the tumour, we first consider the concentration in a 3D rectangular slice of the tumour (**Fig A**). We assume that this 3D domain has a length $l$ and width $w$ and height $h$. Inside this domain is placed a cylindrical fibre. The radius of this fibre is $r_{total}$ and length is $L$, where $r_{total}\ll h$ and $L<l$.


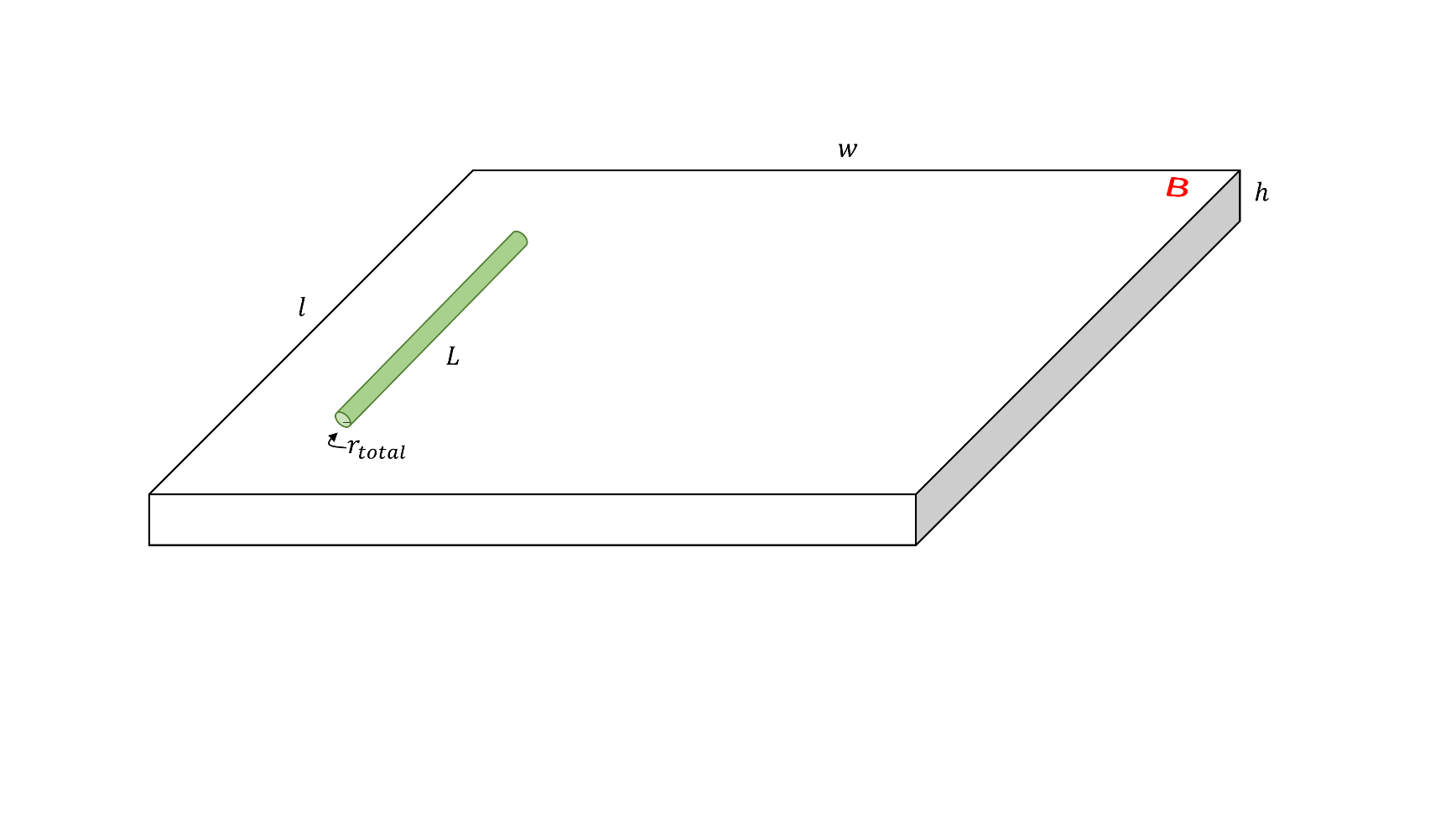


**Fig A. The 3D problem.** The above schematic summarizes the 3D rectangular cross section of the tumour domain considered. Where drug is initially loaded into a cylindrical fibre of length $L$ and radius $r_{total}$. A thin rectangular cross section is taken that contains their fibre and has length $l$, width $w$ and height $h$, where $r_{total}\ll h$. The drug flows out of the fibre into the rectangular domain also called the tumour microenvironment.

To simulate the model, we approximate the 3D problem with a 2D version (**Fig B**), a modeling framework readily used to model cancer growth in agent-based models (ABMs) [1–6]. Since $r_{total}\ll h$, the cylindrical fibre can be represented by a line source in the tumour microenvironment (TME) for the drug outside the fibre. If we denote the concentration of gemcitabine drug in the TME (outside the fibre) by $C(x,y,t)$ inside the domain with boundary $B$ we have

|  | $\frac{\partial C}{\partial t}=D\nabla^{2}C-\lambda C+\delta\left( x-x_{F} \right)J\left( y,t \right)-\sum_{\mathrm{cells}i} \delta\left( x-x_{i} \right)\delta\left( y-y_{i} \right)v_{C}W_{i}C,$ | (TS1) |
| --- | --- | --- |

where $D$ is the diffusion coefficient in the TME, $\lambda$ is the decay rate of the drug, $\delta(x)$ is the Dirac delta function in one-dimension, $(x_{i},y_{i})$ is the $i$th cancer cell’s position in the domain, and $W_{i}$ is the cell’s volume. Cancer cells uptake drug in the domain at a rate $\nu_{c}$. The fibre releases drug into the domain as a line source at position $x=x_{F}, y_{0}<y<y_{0}+L$ with flux $J(y,t)$ where

$$J(y,t)= \left\{ \begin{matrix} -{\frac{2\pi r_{total}}{h}D}_{F}\left( t \right)\frac{\partial F}{\partial r}(r_{total}, y,t) & y_{0}\leq y\leq y_{0}+L \\ 0 & y<y_{0}, y>y_{0}+L \end{matrix} \right.,$$

where $D_{F}(t)$ is the time-dependent diffusion of drug inside the fibre, $F(r,y,t)$ is the concentration of drug inside the fibre at the radial position $r$, $y_{0}$ is the location of the bottom of the fibre and $h$ is the depth of the thin rectangular region that we are modelling the concentration of drug within (**Fig A**). The diffusivity of the drug, $D_{F}(t)$, is modeled by the function

|  | $D_{F}\left( t \right)=\frac{k}{t+\epsilon}+D_{const},$ | (TS2) |
| --- | --- | --- |

where $k$ controls the decay rate to the constant decay rate from the fibre (i.e. how quickly the fibre swells), $D_{const}$ is the constant decay rate from the fibre and $\epsilon$ is a tuning constant to avoid the singularity at $t=0$. In the derivation here we assume the fibre is placed vertically (parallel to the $y$-axis); however, it is possible to derive the equivalent form for the fibre being placed horizontally. We assume the diffusion of drug inside the fibre can be modeled as radially symmetric and that diffusion in the radial direction is significantly faster than along the fibre (given $r_{total}\ll L$). This gives

|  | $\frac{\partial F}{\partial t}=D_{F}\left( t \right)\frac{1}{r}\frac{\partial}{\partial r}\left( r\frac{\partial F}{\partial r} \right).$ | (TS3) |
| --- | --- | --- |


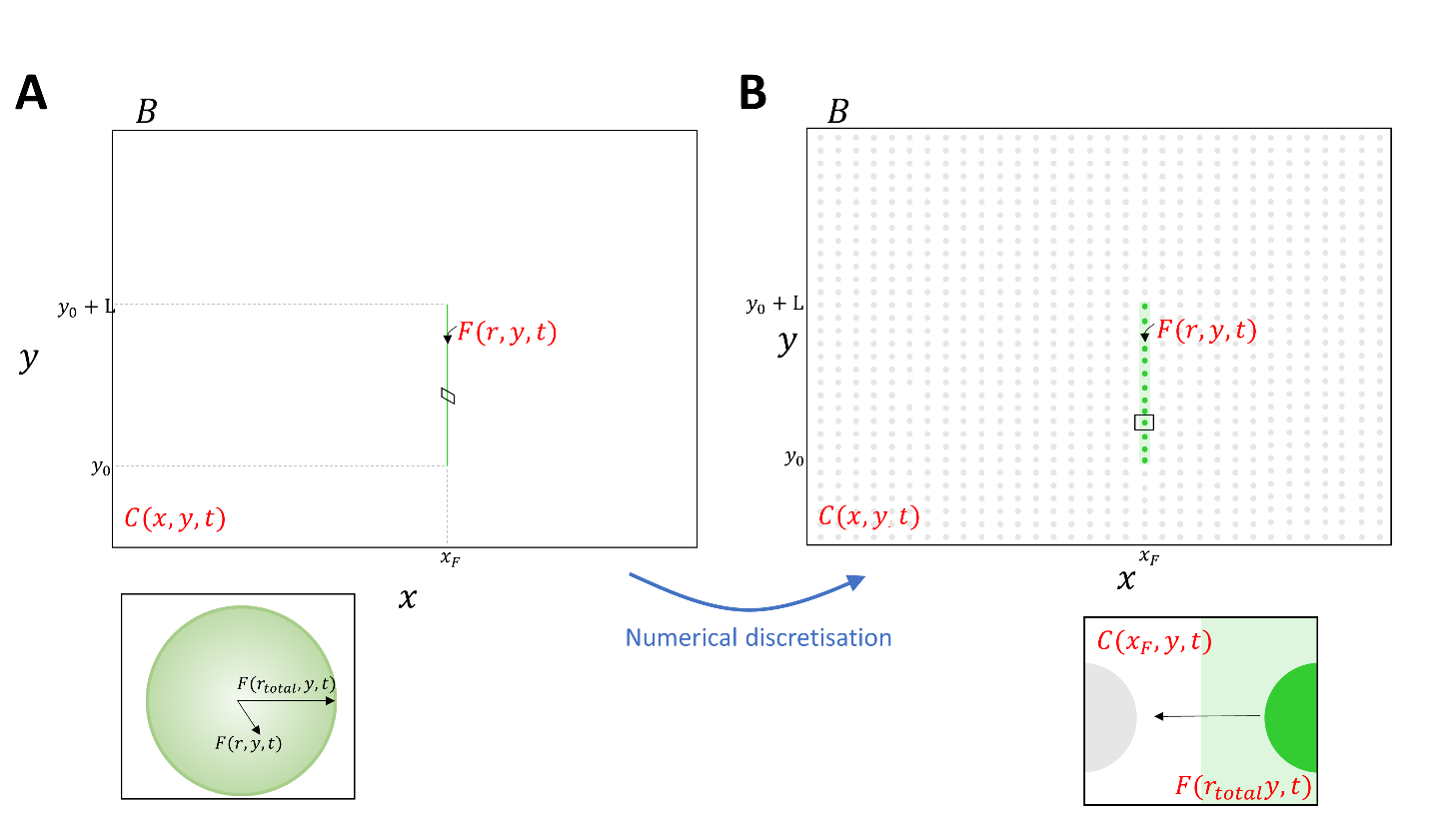


**Fig B. Illustration for the 2D problem in the continuous and FVM discretisation setting.** (A) The concentration of drug in the TME (i.e. inside the region bounded by $B$) is given by $C(x,y,t)$. We consider a simplification of the 3D problem presented in **Fig A** by modelling the fibre as a line-source (see **Eqs. (TS1)-(TS3)**). The concentration of drug inside the fibre is given by $F(r,y,t)$. The bottom end of the fibre is placed at $(x_{F},y_{0}$) and the fibre has length $L$. The drug inside the fibre is modelled as a concentration $F(r,y,t)$ at radius $r$ and cross section $y$ (see zoomed in insert). (B) Using a FVM discretisation we discretise the domain into voxels and consider the fibre as a set of point source terms (see the zoomed in insert).

No-flux boundary conditions on $B$, the exterior of the TME, are imposed:

$$\frac{\partial C}{\partial\vec{n}}=0$$

where $\vec{n}$ is the outward unit normal on the boundary $B$. The boundary condition for the edge of the fibre comes from matching with the concentration in the surrounding TME:

$$F\left( r_{total},y,t \right)=C\left( x_{F},y,t \right).$$

In the case of a fibre implantation, all drug in the domain is initially situated in the fibre:

$$F\left( r,y,0 \right)=\frac{C_{0}}{\pi r_{total}^{2}L}, C\left( x,y,0 \right)=0,$$

Where $C_{0}$ is the amount of drug in $\mu g$ and there is no drug initially in the domain $B$. In the case of single point free-drug injections in the absence of the fibre this changes to

$$C\left( x,y,0 \right)=\sum_{inj} \frac{C_{0}}{hN_{inj}}\delta\left( x-x_{inj} \right)\delta\left( y-y_{inf} \right),$$

where $(x_{inj},y_{inj})$ are the points where the free injections take place and $N_{inj}$ is the total number of injections. In this document, we derive the discretisation for numerically simulating the fibre implantation, and the single free-drug injection, which is a simplification of this, is not described.

In the following sections, we present the method for approximating the system of partial differential equations (PDEs) describing the gemcitabine concentration in the TME, $C(x,y,t)$, and inside the fibre, $F(r,y,t)$ using a Finite Volume Method (FVM) framework. We first discretise the spatial component of the system of PDE for the concentration in the TME (**Eq. (TS1)**) using FVM. We then use FVM to approximate the boundary conditions for the outer boundary of the TME. This discretisation also takes into account the line source from the fibre. The drug concentration inside the fibre is approximated using a FVM of a radial diffusion PDE (**Eq**. **(TS3)**).

We note that many problems in cancer biology and tissue engineering have diffusive processes occurring at relatively fast time scales compared to cellular processes and modelers have captured this by implemented multiple time scales or time steps [7–10]. As such, we consider two timescales (**Fig C**) in our time-discretisation of the system: a diffusive biotransport time step $\Delta t=1 \min$ and a cellular uptake time scale $\Delta t_{cells}=1\mathrm{hour}$. The drug diffusion, decay and release from the fibre is all considered to occur on the diffusive time step $\Delta t$ and the cell uptake term is evaluated on the cell uptake time scale $\Delta t_{cells}$. Mathematically, this time scale separation allows us to hold cell positions fixed (quasi-static) when updating the PDE solutions, and then hold the drug field while updating the cell positions and cell uptake, death or proliferation. To advance both the cellular and diffusive processes in time we use a forward Euler approximation.


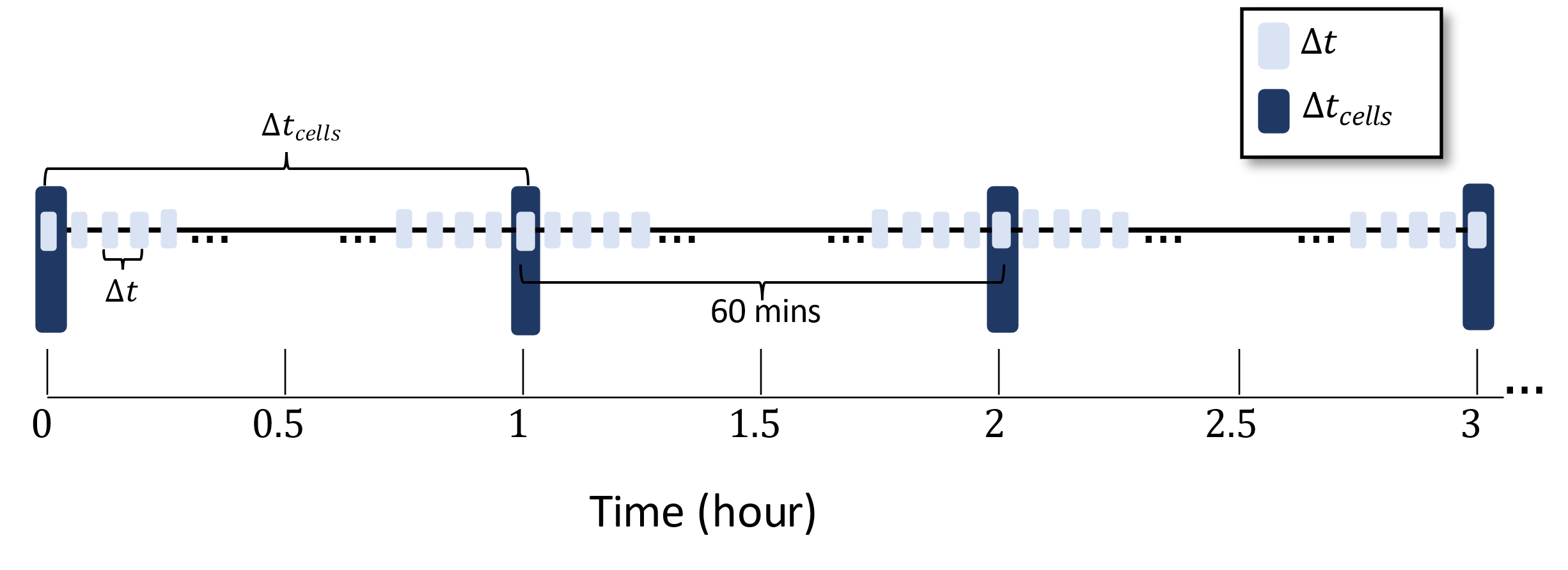


**Fig C. Illustration for the time-stepping in the VCBM-PDE**. Diffusive processes were modelled as occurring on a time step of $\Delta t$. Cellular processes occurred on longer time steps of $\Delta t_{cells}$.

# TS2 Numerical approximation for gemcitabine concentration in the tumour microenvironment

We can solve PDEs numerically using FVM. There are many examples of this type of method applied in modeling cancer growth and treatment [11–19]. We introduce the numerical approximation to the system of equations in **Eqs. (TS1)-(TS3)** in the absence of uptake by cells, which is included in the time discretisation (**Section TS.4**).

## TS2.1 Drug diffusion in 2-dimensions in the TME

In **Eq. (TS1)**, we described the concentration $C\left( x,y,t \right)$ of gemcitabine drug in the TME. Here we show how to discretise this equation using FVM. Rearranging **Eq. (TS1)** gives

$$0=\frac{\partial C\left( x,y,t \right)}{\partial t}+\nabla\cdot\left( -D\nabla C\left( x,y,t \right) \right)+\lambda C\left( x,y,t \right)-S(x,y,t)$$

Where $S(x,y,t)$ represents the source term due to the fibre (as the cell uptake occurs on a different time scale we will describe its inclusion separately). We sub-divide the two-dimensional spatial domain $B$ into $N\times K$ rectangles $b_{i,j}$of size $\Delta x \times\Delta y$and centre $x_{i},y_{j}$, with

$$\Delta x=\frac{l}{N},\Delta y=\frac{w}{K}, x_{i}=\Delta x\left( i-\frac{1}{2} \right), y_{j}=\Delta y\left( j-\frac{1}{2} \right), i=1,\ldots, N, j=1, \ldots, K.$$

For a particular rectangle $b_{i,j}$ in the discretisation we take the integral over the total area:

|  | $0=\iint_{b_{i,j}} \frac{\partial C}{\partial t}dydx+ \iint_{b_{i,j}} \nabla\cdot\boldsymbol{J} dydx+\lambda\iint_{b_{i,j}} C dydx- \iint_{b_{i,j}} S dydx,$ | (TS4) |
| --- | --- | --- |

where

|  | $\boldsymbol{J}= -D\nabla C\left( x,y,t \right).$ | (TS5) |
| --- | --- | --- |

The control volume averaged concentrations $\overline{C}_{i,j}\left( t \right)$ and $\overline{S}_{i,j}\left( t \right)$ are defined to be

$$\overline{C}_{i,j}\left( t \right)=\frac{1}{\Delta x\Delta y}\iint_{b_{i,j}} C\left( x,y,t \right)dydx, \overline{S}_{i,j}\left( t \right)=\frac{1}{\Delta x\Delta y}\iint_{b_{i,j}} S\left( x,y,t \right)dydx.$$

From these definitions, and applying the divergence theorem, or Green’s theorem in 2D,

|  | $0= \frac{d\bar{C}_{i,j}(t)}{dt}+\frac{1}{\Delta x\Delta y}\oint_{\Gamma_{i,j}} \boldsymbol{J\cdot}\hat{n}dl+\lambda\overline{C}_{i,j}\left( t \right)+\overline{S}_{i,j}(t),$ | (TS6) |
| --- | --- | --- |

where $\hat{n}$ is the outward unit normal on the boundary $\Gamma_{i,j}$of the control volume $b_{i,j}$. As the control volumes are rectangular, we consider four distinct faces for the boundary $\Gamma_{i,j}=\{\Gamma_{e},\Gamma_{w}, \Gamma_{n}, \Gamma_{s}$}, where $e,w,n$ and $s$ stand for east face, west face, north face and south face, respectively; see **Fig D** for an illustration of the domain. We then use a midpoint quadrature approximation for the line integral of each face in **Eq. (TS6)**, which gives

|  | $0= \frac{d\bar{C}_{i,j}(t)}{dt}+\frac{1}{\Delta x\Delta y}\left( \Delta y\left( J_{e}-J_{w} \right)+\Delta x\left( J_{n}-J_{s} \right) \right)-\lambda\overline{C}_{i,j}+\overline{S}_{i,j},$ | (TS7) |
| --- | --- | --- |

where

$$J_{e}= -D\left( \frac{\partial C}{\partial x} \right)_{e}, J_{w}=-D\left( \frac{\partial C}{\partial x} \right)_{w}, J_{n}=-D\left( \frac{\partial C}{\partial y} \right)_{n}, J_{s}=-D\left( \frac{\partial C}{\partial y} \right)_{s}.$$

We now drop the use of the bar above $C_{i,j}$ for simplicity (i.e. $\bar{C}_{i,j}=C_{i,j})$ and using a central difference approximation for these partial derivatives we have

$$J_{e}\approx-D \left( \frac{C_{i+1,j}-C_{i,j}}{\Delta x} \right), J_{w}\approx-D\left( \frac{C_{i,j}-C_{i-1,j}}{\Delta x} \right), J_{n}\approx-D\left( \frac{C_{i,j+1}-C_{i,j}}{\Delta y} \right), J_{s}\approx-D\left( \frac{C_{i,j}-C_{i,j-1}}{\Delta y} \right)$$

Assuming for simplicity that the control volumes are square, that is, $\Delta x=\Delta y=\Delta$, then substituting into **Eq. (TS7)** and rearranging gives

|  | $\frac{dC_{i,j}}{dt}=\frac{D}{\Delta^{2}}\left( C_{i-1,j}+C_{i+1,j}+C_{i,j-1}+C_{i, j+1}-4C_{i,j} \right)-\lambda C_{i,j}+S_{i,j}.$ | (TS8) |
| --- | --- | --- |

This gives the equation for approximating the change in concentration at node $(i,j)$ given the approximate concentrations at the neighbouring nodes.

## TS2.2 Outer TME boundary conditions

We assume no-flux boundary conditions on the outer domain $B$. That is

$$\frac{\partial C}{\partial\hat{n}}\left( t,\left( x,y \right)\in B \right)=0$$

**
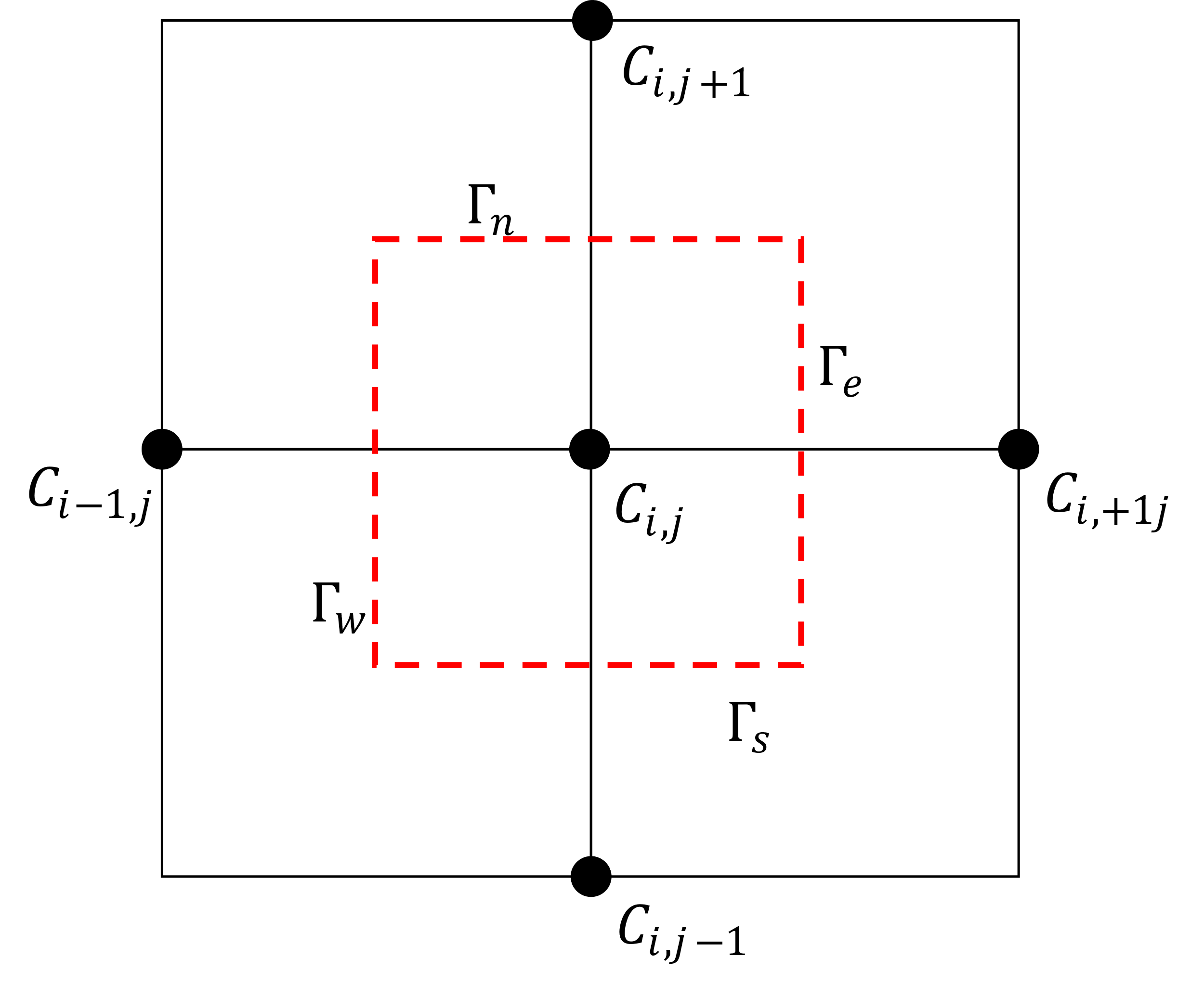
**

**Fig D. Schematic depicting the domain discretisation for the FVM.** The concentration of drug at node $(i,j)$ in the discretised domain is given by $C_{i,j}(t)$. We used a FVM to approximate the diffusion across the domain. There are 4 faces of the control volume at node $C_{i,j}$, given by $\Gamma_{e}, \Gamma_{w}, \Gamma_{n}$ and $\Gamma_{s}$, where these are the east, west, north and south faces depicted on the schematic. We consider drug fluxes through these faces from the neighbouring nodes $C_{i-1,j}, C_{i+1,j}, C_{i,j-1}$ and $C_{i, j+1}$.

Where $\hat{n}$ is the unit normal on $B$. Let $B_{e}, B_{w}, B_{n},$ and $B_{s}$describe the east, west, north and south faces of the domain (see **Fig E**), where $B_{e}\cup B_{w}\cup B_{n}\cup B_{s}=B$, and let $B_{e}\cap B_{n}, B_{e}\cap B_{s}, B_{w}\cap B_{n}$ and $B_{w}\cap B_{n}$ be the corners of the domain. Then the no-flux boundary conditions can be considered by

|  | $\left( \frac{\partial C}{\partial x} \right)_{B_{e}}=0, \left( \frac{\partial C}{\partial x} \right)_{B_{w}}=0, \left( \frac{\partial C}{\partial y} \right)_{B_{n}}=0, \left( \frac{\partial C}{\partial y} \right)_{B_{s}}=0,$ $\left( \frac{\partial C}{\partial x} \right)_{B_{e}\cap B_{n}}=\left( \frac{\partial C}{\partial y} \right)_{B_{e}\cap B_{n}}=0, \left( \frac{\partial C}{\partial x} \right)_{B_{e}\cap B_{s}}=\left( \frac{\partial C}{\partial y} \right)_{B_{e}\cap B_{s}}=0,$ $\left( \frac{\partial C}{\partial x} \right)_{B_{w}\cap B_{n}}=\left( \frac{\partial C}{\partial y} \right)_{B_{w}\cap B_{n}}=0, \left( \frac{\partial C}{\partial x} \right)_{B_{w}\cap B_{s}}=\left( \frac{\partial C}{\partial y} \right)_{B_{w}\cap B_{s}}=0.$  $.$ | (TS9) |
| --- | --- | --- |

We can derive finite volume approximations for these using **Eq.(TS7)**. We provide an example for this derivation on the boundary $B_{e}$ and follow an equivalent process for the remainder of the boundary conditions given above in **Eq. (TS9)**. Given the no-flux boundary, the flux through the east face $J_{e}=0$, so

$$0= \frac{dC_{N,j}\left( t \right)}{dt}+\frac{1}{\Delta x\Delta y}\left( \Delta y\left( -J_{w} \right)+\Delta x\left( J_{n}-J_{s} \right) \right)-\lambda C_{N,j}+S_{N,j}.$$

Using a central difference approximation for the partial derivatives we have

$$\frac{dC_{N,j}}{dt}\approx\frac{D}{\Delta x\Delta y}\left[ -\Delta y\left( \frac{C_{N,j}-C_{N-1,j}}{\Delta x} \right)+\Delta x\left( \frac{C_{N,j+1}-C_{N,j}}{\Delta y} \right)-\Delta x\left( \frac{C_{N,j}-C_{N+1,j}}{\Delta y} \right) \right]-\lambda C_{N,j}+S_{N,j},$$

Which assuming $\Delta x=\Delta y=\Delta$, simplifies to

$$\frac{dC_{N,j}}{dt}=\frac{D}{\Delta^{2}}\left[ C_{N-1,j}+C_{N,j+1}+C_{N,j-1}-3C_{N,j} \right]-\lambda C_{N,j}+S_{N,j}. \forall j=1,\ldots K$$

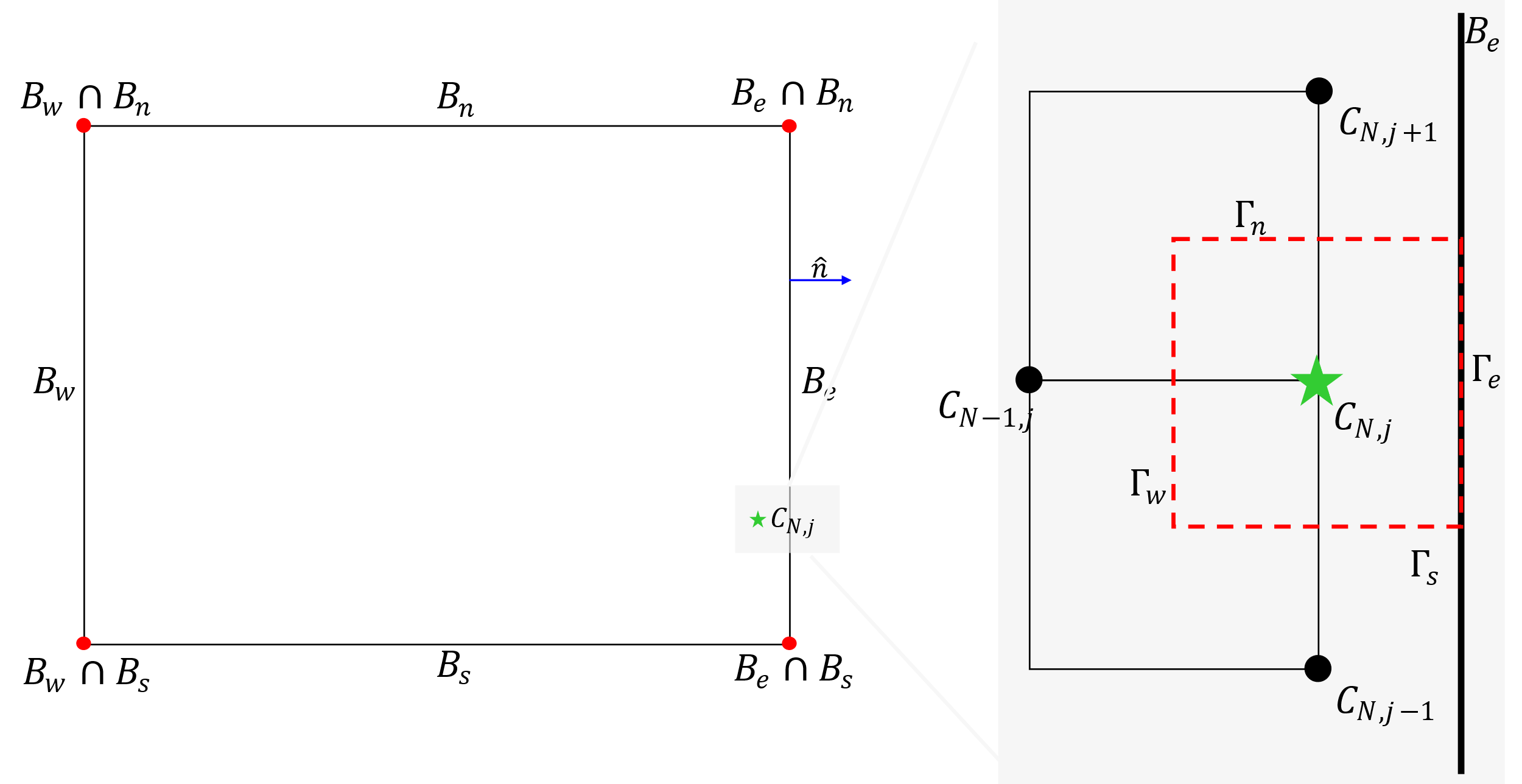


**Fig E. Schematic depicting the boundary of the domain** $\boldsymbol{B}$ **defined as the boundary of the tumour microenvironment (TME).** There are four edges to the boundary of the domain, defined as $B_{n}, B_{e}, B_{s},$and $B_{w}$ (north edge, east edge, south edge and west edge). The corners of the boundary are the intersections of these edges $B_{e}\cap B_{n}, B_{e}\cap B_{s}, B_{w}\cap B_{n}$ and $B_{w}\cap B_{s}$. We consider these edges and corners to be defined by the face of voxel nodes in the FVM discretisation and the no flux boundary conditions to be applied everywhere. In other words, the derivative of $B$ on the outward unit normal, $\hat{n}$, is equal to zero. In the grey insert, a voxel adjacent to the east boundary node $C_{N,j}$ are shown. The east face of this voxel aligns with the boundary wall $B_{e}$. The derivation of the FVM approximation for the drug concentration at this node is given in **Eq. (TS9)**.

At a corner, two fluxes vanish. For example, at the top right corner ($i=N,j=K$),

$$\frac{d\overline{C}_{N,K}}{dt}=\frac{D}{\Delta^{2}}\left[ \overline{C}_{N-1,K}+\overline{C}_{N,K-1}-2\overline{C}_{N,K} \right]-\lambda\overline{C}_{N,K}+\overline{S}_{N,K},$$

with a similar formula for each of the other corners. For further depiction of the boundary conditions, see **Fig E .**

## TS2.3 Drug diffusion within the fibre

Polymeric fibres are known to release drug slowly over time through degradation and swelling. To model this, we consider that drug will slowly diffuse out of the fibre over time (**Eq. (TS3)**). We assume for simplicity that the fibre is homogeneous, and we can model drug diffusion and movement within the fibre using a radially symmetric PDE. **Eq. (TS3)** can be approximated by a system of ODEs, similar to the FVM described in the previous section. It is also assumed that the fibre length $L$ is an integer multiple of $\Delta y$and that each control volume that contains the fibre contains a length of section $\Delta y$ (see **Fig** 5). Let $F_{m,j}(t)$ be the concentration ($\mu g/\mu l$) of drug in the $m$th annulus of a cylindrical fibre cross section (**Fig 1**A Main Text), where $m=1,2,\ldots,M$, and $C_{i,j}$be the concentration of drug outside the fibre in control volume $b_{i,j}$which contains the $j$th section of the fibre. The equivalent ODE model for diffusion of the drug between annuli is

|  | $\frac{dF_{1,j}}{dt}=2\frac{D_{F}\left( t \right)}{\Delta r^{2}}{(F}_{2,j}-F_{1,j}),$ | (TS10) |
| --- | --- | --- |
|  | $\frac{dF_{m,j}}{dt}=\frac{D_{F}\left( t \right)}{\Delta r^{2}}\left( \frac{m\Delta r}{\left( m-\frac{1}{2} \right)\Delta r}\left( F_{m+1,j}-F_{m,j} \right)-\frac{\left( m-1 \right)\Delta r}{\left( m-\frac{1}{2} \right)\Delta r}\left( F_{m,j}-F_{m-1,j} \right) \right),$ | (TS11) |
|  | $\frac{dF_{M,j}}{dt}=\frac{D_{F}\left( t \right)}{\Delta r^{2}}\left( \frac{2M\Delta r}{\left( M-\frac{1}{2} \right)\Delta r}\left( C_{i,j}-F_{M,j} \right)-\frac{\left( M-1 \right)\Delta r}{\left( M-\frac{1}{2} \right)\Delta r}\left( F_{M,j}-F_{M-1,j} \right) \right),$ | (TS12) |

where $\Delta r=r_{total}M$. The flux out of the fibre into the surrounding control volume $b_{i.j}$ is then

|  | $S_{i,j}=\frac{2\pi r_{total}}{h\Delta}\frac{\left( F_{M,j}-C_{i,j} \right)}{\Delta r/2}.$ | (TS13) |
| --- | --- | --- |

## TS2.4 Cell uptake and time discretisation

Similar to work by Ghaffarizadeh *et al.* [7], we assume cell uptake happens on a much slower time scale, $\Delta t_{cells}$, than diffusion and decay, $\Delta t$, see **Fig C**. As such, we solve the FVM system of ODEs describing the drug concentration and then update the uptake of cells every $\Delta t_{cells}=1$hour. Using a forward Euler scheme, where $C_{i,j}^{n}=C_{i,j}(t=n\Delta t)$,

$$C_{i,j}^{(n+1)}=C_{i,j}^{(n)}+\Delta t\frac{dC_{i,j}^{(n)}}{dt},$$

where $\frac{dC_{i,j}^{n}}{dt}$ are the expressions from the preceding section that include diffusion, decay and source terms; and when $n$ is an integer multiple of $\Delta t_{cells}/\Delta t$, we update the concentration with the cell uptake terms, at the same time as updating the cells, before recommencing the diffusive process:

$$C_{i,j}^{(n)}\mapsto C_{i,j}^{(n)}+\sum_{k} \chi_{i,j,k}{\frac{\Delta t_{cells}}{\Delta^{2}}W}_{k}\nu_{c}C_{i,j}^{(n)}, n=integer \times\frac{\Delta t_{cells}}{\Delta t},$$

where $\chi_{i,j,k}=1$ when cell $k$ is in the rectangle $b_{i,j}$, and zero otherwise.

# TS3. Fibre release functions

To analyse the effect of the drug release from the fibre, we investigate four different release profiles: constant release, exponential release, sigmoidal Emax release and sigmoidal Imax release. To do this, we simplified the model for the concentration of drug inside the fibre $F(x,y,t)$ to simply to be $F(t)$, where the release of the fibre no longer depended on the radial concentration of drug or the location of the fibre. In this way, we could consider purely the release profile and its impact on the treatment efficacy.

For each release profile, to conserve the maximum amount of drug possible to release and confirm that no single release profile released more than the total amount in the original fibre (**Fig 2**B), we set $F\left( 0 \right)=C_{max}$. Note, some release profiles released less drug than the original fibre as their release profiles were slower in the time frame of the simulation.

For the simple constant release of drug from the fibre, we used

$$\frac{dF}{dt}=\left\{ \begin{matrix} -\gamma& F>0 \\ 0 & F\leq0 \end{matrix} \right.$$

where $\gamma$ is the release rate of the drug. In the numerical scheme, this meant

$$S_{i,j}=-\frac{dF}{dt}.$$

As a constant release can result in negative drug concentration inside the fibre we introduced the above piecewise constraint. We next considered an exponential release:

$$\frac{dF}{dt}=-\gamma F, S_{i,j}=\gamma F.$$

For the sigmoidal Emax and Imax curves [20–22], we used the following forms

$$\frac{dF}{dt}=\gamma\frac{F}{F+\eta}F, \frac{dF}{dt}=\gamma\frac{\eta}{F+\eta}F,$$

where $\eta$ is the half-effect concentration (i.e. the concentration at which half of the maximum possible release rate is achieved), see **Fig F.**


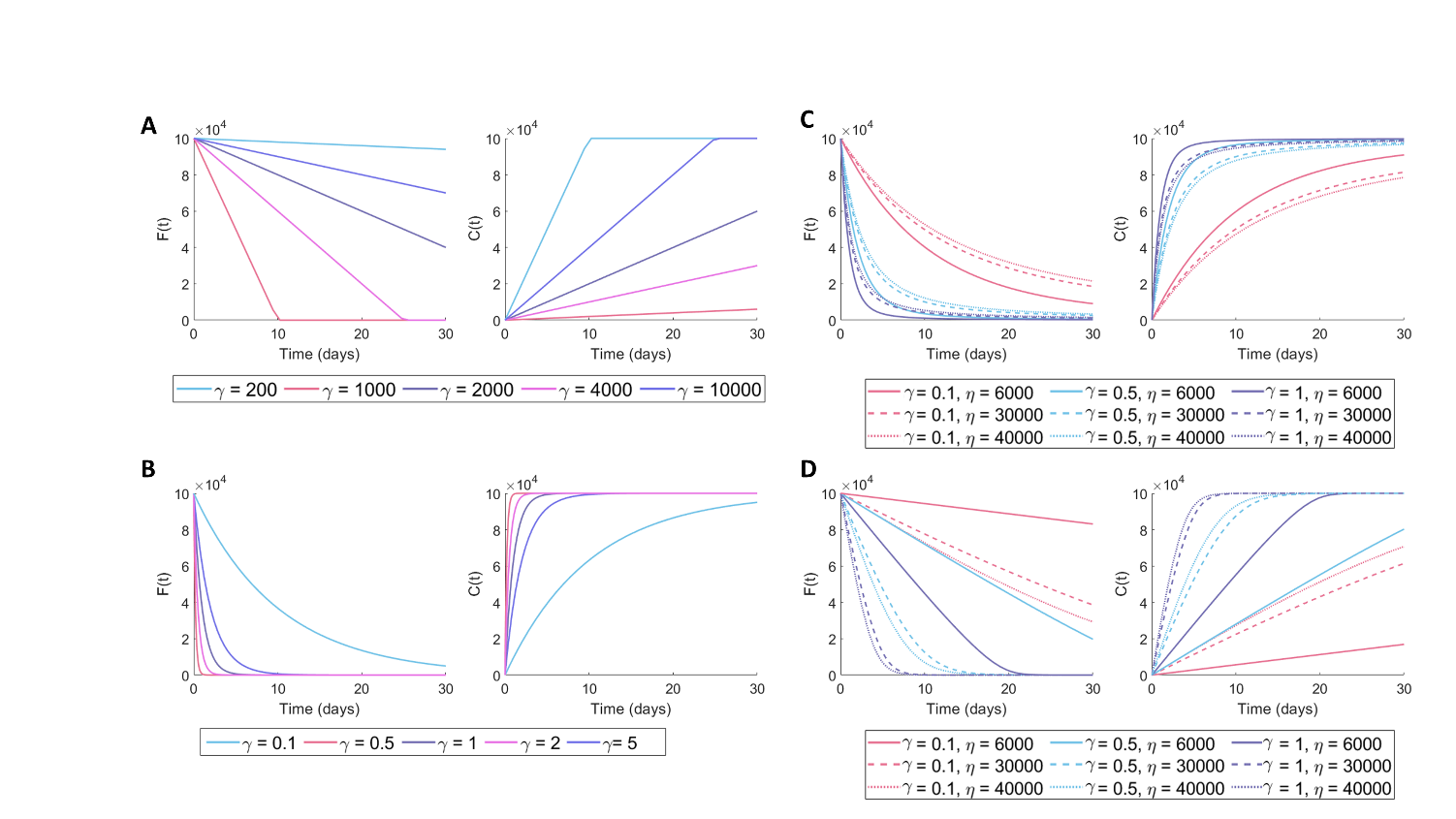
 **Fig F. Effect of** $\boldsymbol{\gamma, \eta}$ **on different fibre release profiles.** $F$ is the total concentration inside the fibre and $C$ is the total concentration outside the fibre. We remove all decay, uptake effects from the TME so we can confirm a confirmation of the drug concentration. The colour of the line indicates a different value of $\gamma,$while a different line style indicates a new value of $\eta.$ For all four release profiles, increasing the value of $\delta$ increases the speed of release and thereby decreases the time taken for the entire dose to be given (A) Concentration of injection inside the fibre over 33 days with a constant release rate, $\frac{dF}{dt}=-\gamma$. (B) Concentration of injection inside the fibre over 33 days with a exponential release rate, $\frac{dF}{dt}=-\gamma F.$ (C) Concentration of injection inside the fibre over 33 days with a sigmoidal release rate, $\frac{dF}{dt}=-\frac{\gamma F}{F+\eta}F$. Increasing $\eta$ slows the release. For a different sigmoidal release profile (D), $\frac{dC_{in}}{dt}=-\frac{\gamma\eta}{F+\eta}F$, increasing $\eta$ speeds up the release

# TS4 References

1. Bacevic K, Noble R, Soffar A, Ammar OW, Boszonyik B, Prieto S, et al (2017) Spatial competition constrains resistance to targeted cancer therapy. Nat Commun 8:1995

2. Gilmore AC, Flaherty SJ, Somasundaram V, Scheiblin DA, Lockett SJ, Wink DA, et al (2021) An in vitro tumorigenesis model based on live-cell-generated oxygen and nutrient gradients. Commun Biol 4:1–13

3. Enderling H, Hlatky L, Hahnfeldt P (2009) Migration rules: Tumours are conglomerates of self-metastases. Br J Cancer 100:1917–1925

4. Brüningk SC, Ziegenhein P, Rivens I, Oelfke U, Haar G ter (2019) A cellular automaton model for spheroid response to radiation and hyperthermia treatments. Sci Rep 9:1–12

5. Wang Z, Butner JD, Kerketta R, Cristini V, Deisboeck TS (2015) Simulating cancer growth with multiscale agent-based modeling. Semin Cancer Biol 30:70–78

6. Pourhasanzade F, Sabzpoushan SH (2021) A New Mathematical Model for Controlling Tumor Growth Based on Microenvironment Acidity and Oxygen Concentration. Biomed Res Int. https://doi.org/10.1155/2021/8886050

7. Ghaffarizadeh A, Heiland R, Friedman SH, Mumenthaler SM, Macklin P (2018) PhysiCell: An open source physics-based cell simulator for 3-D multicellular systems. PLoS Comput Biol 14:e1005991

8. Macklin P, Lowengrub JS (2008) A new ghost cell/level set method for moving boundary problems: application to tumor growth. J Sci Comput 35:266–299

9. Gong C, Milberg O, Wang B, Vicini P, Narwal R, Roskos L, et al (2017) A computational multiscale agent-based model for simulating spatio-temporal tumour immune response to PD1 and PDL1 inhibition. J R Soc Interface 14:20170320

10. Schuetz TA, Becker S, Mang A, Toma A, Buzug TM (2013) Modelling of glioblastoma growth by linking a molecular interaction network with an agent-based model. Math Comput Model Dyn Syst 19:417–433

11. Pasdunkorale A J, Turner IW (2005) A second order control-volume finite-element least-squares strategy for simulating diffusion in strongly anisotropic media. J Comput Math 1–16

12. Chaudhry QA, Abbas A, Noor A, Asif M (2019) In silico modeling for the risk assessment of toxicity in cells. Comput \& Math with Appl 77:1541–1548

13. Shakeri F, Dehghan M (2011) The finite volume spectral element method to solve Turing models in the biological pattern formation. Comput \& Math with Appl 62:4322–4336

14. Andasari V, Gerisch A, Lolas G, South AP, Chaplain MAJ (2011) Mathematical modeling of cancer cell invasion of tissue: biological insight from mathematical analysis and computational simulation. J Math Biol 63:141–171

15. Hubbard ME, Byrne HM (2013) Multiphase modelling of vascular tumour growth in two spatial dimensions. J Theor Biol 316:70–89

16. Eymard R, Gallouët T, Herbin R (2000) Finite volume methods. Handb Numer Anal 7:713–1018

17. Khalid S, Chaudhry QA (2019) Quantitative analysis of cancer risk assessment in a mammalian cell with the inclusion of mitochondria. Comput \& Math with Appl 78:2449–2467

18. Ain K, Wibowo RA, Soelistiono S (2017) Modeling of electrical impedance tomography to detect breast cancer by finite volume methods. In: J. Phys. Conf. Ser. p 12001

19. Storey KM, Jackson TL (2021) An Agent-Based Model of Combination Oncolytic Viral Therapy and Anti-PD-1 Immunotherapy Reveals the Importance of Spatial Location When Treating Glioblastoma. Cancers (Basel) 13:5314

20. Upton RN, Mould DR (2014) Basic concepts in population modeling, simulation, and model-based drug development: Part 3-introduction to pharmacodynamic modeling methods. CPT Pharmacometrics Syst Pharmacol 3:1–16

21. Gabrielsson J, Andersson R, Jirstrand M, Hjorth S (2019) Dose-response-time data analysis: an underexploited trinity. Pharmacol Rev 71:89–122

22. Prinz H (2010) Hill coefficients, dose--response curves and allosteric mechanisms. J Chem Biol 3:37–44
