# Supplementary material for: Examining the efficacy of localised gemcitabine therapy for the treatment of pancreatic cancer using a hybrid agent-based model
Source: PLoS Comput Biol. 2023 Jan 17;19(1):e1010104. doi: 10.1371/journal.pcbi.1010104 (PMC9891514; doi:10.1371/journal.pcbi.1010104)
Supplement: S1 Code Documentation — Corresponds to the code on Github. (DOCX) [file pcbi.1010104.s003.docx]

**Code documentation: Examining the efficacy of drug-loaded polymers in the treatment of pancreatic cancer using a hybrid agent-based model.**

Adrianne L. Jenner^1^, Wayne Kelly^2^, Michael Dallaston^1^, Robyn Araujo^1^, Isobelle Parfitt^1^, Dominic Steinz^3,4^, Pantea Pooladvand^5^, Peter S. Kim^5^, Samantha J Wade^6^, Kara L. Vine^6,7^,

Contents

[1. Introduction 2](#_Toc95139006)

[2. The QuickStart: running a simulation 2](#_Toc95139007)

[2.1 Running a simulation in MATLAB 2](#_Toc95139008)

[2.2 Regenerating the MATLAB interface to the C++ library 2](#_Toc95139009)

[3. Overall C++ code structure 3](#_Toc95139010)

[3.1 Description of Model.cpp and Model.h 3](#_Toc95139011)

[3.2 Description of Params.hpp 3](#_Toc95139012)

[3.3 Description of Pancreas.hpp 3](#_Toc95139013)

[3.4 Description of Cell.hpp 3](#_Toc95139014)

[3.5 Description of Helper.hpp 3](#_Toc95139015)

[3.6 Description of delaunator.hpp 3](#_Toc95139016)

[3.7 Description of Diffuse.hpp 3](#_Toc95139017)

[4. Cell agents 4](#_Toc95139018)

[5. Diffusion script 5](#_Toc95139019)

[6. Delaunay triangulation 6](#_Toc95139020)

[7. Pancreas 6](#_Toc95139021)

[8. Plotting 8](#_Toc95139022)

[8.1 Plotting in MATLAB 8](#_Toc95139023)

[8.2 Plotting in C++ 8](#_Toc95139024)

[9. References 8](#_Toc95139025)

# Introduction

In this document, an outline of the code structure for the Voronoi Cell-Based Model-partial differential equation (VCBM-PDE) is given as well as a general guide to simulating and running the code. Code can be accessed on github: <https://github.com/AdrianneJennerQUT/hybrid-VCBM-of-gemcitabine-and-pancreatic-cancer>.

Full details for the mathematical theory behind different aspects of the model can be found in either the main text, **S1 Technical Supplementary Information** or in the previous publication of the model by Jenner *et al.* [1].

The underlying VCBM-PDE model is written in C++ and the code includes a Matlab extension so that the model can be simulated, and results accessed through MATLAB. This is done through using MATLAB’s clibgen function which creates a MATLAB interface to the C++ library storing the VCBM-PDE model. It is possible to run the VCBM-PDE fully through C++ using Visual Studio or otherwise, however, we do not include instructions for this here.

In this guide, we will detail how to call the VCBM-PDE through MATLAB and simulate the model for different parameter values. We will also detail how to make changes to the C++ library and recreate the MATLAB interface using clibgen. After this, we will talk through the structure of the code so that future users can make changes to the VCBM-PDE.

# The QuickStart: running a simulation

## Running a simulation in MATLAB

To run a simulation of the VCBM-PDE first download the source code as a .zip file and unzip. There are two options for running the code, the first is through MATLAB and the second is through C++ directly, using Visual Studio or otherwise. Below describes how to run the code through MATLAB.

You will need a version of MATLAB 2021a or onwards to be able to run the code. In MATLAB, navigate to the unzipped folder. Note that the code has been segregated into either control tumour growth code (“TumourModelControl”), tumour growth under a injection/s of drug (“TumourModelSingleInjectionCPP”) and tumour growth under fibre treatment (“TumourModelFibreCPP”). For these three scenarios, all the C++ code is stored in the folder “TumourModel”. The MATLAB command scripts which call the C++ code are stored in the main directory and the C++ Matlab interface library is in the folder “Model” and is called ModelInterface.dll. To simulate control tumour growth, no treatment, run commands_controlgrowthsimulation.m.

Parameter values can then be changed in VCBM-PDE the model by simply updating them in the MATLAB script and re-running, this includes the initial conditions of the simulation, such as initial tumour size at treatment and initial drug concentration and location.

If there are issues running the pre-defined MATLAB interface with the C++ library, or you wish to change the underlying C++ code and re-run, please use the instructions in the following section.

## Regenerating the MATLAB interface to the C++ library

In some instances, you may need to regenerate the MATLAB interface to the C++ library. This might occur if you are using as OSX machine or if C++ is not already integrated with MATLAB on your machine (try mex -setup CPP -v to check the setup on your machine). To regenerate the MATLAB interface with C++, first delete the folder “Model” and the code defineModel.m and ModelData.xml. In MATLAB’s command line, run

clibgen.generateLibraryDefinition(“TumourModel\Model.cpp”)

This will generate a .m and .mlx file define the interface for MATLAB and the VCBM-PDE C++ code. Delete the defineModel.mlx script. Open the file defineModel.m and uncomment any commented C++ code functions. Replace all places where it says <SHAPE> to 1. In the MATLAB command line then run build(defineModel).

After the project builds, there should be a hyperlink that will add the interface file to the folder path, click on this or add this path manually to your MATLAB. It should now be possible to run commands_controlgrowthsimulation.m.

# Overall C++ code structure

The C++ code is structured into sections which are called through the main Model.cpp. The following details the function of the different .hpp files and how they integrate together:

## Description of Model.cpp and Model.h

In this script, we define the main functions of the VCBM-PDE simulation and call the various aspects of the model. This script contains different options for simulating the model, and these functions are read and can be called through MATLAB.

Pancreas* SeedAndGrowToStartVolumeM(…, startVolume): simulates the tumour growth model from one cell until it reaches the value in startVolume for the parameter values p0, psc, dmax, gage, page. For example, setting startVolume to be 200 the model will run from one cell until it reaches a volume of startVolume. It will then return the pancreas object Pancreas, which is the state of the model at the time it stopped the simulation, i.e. when the tumour volume reached 200.

void SimulateWholeExperiment(…, timeSteps,): Once a starting volume has been reached, the pancreas object can be sent to the VCBM-PDE and a whole experiment for some number of timeSteps can be simulated. To complete a whole simulation experiment, the model is iterated through a for loop, where a single day is simulated using SimulateOneDay().

There are other functions contained in the Model.cpp that follow a similar layout to what is described above. The header file Model.h is used to declare the C++ functions.

## Description of Params.hpp

In this script, we initialise all the parameters in the model and set any pre-defined fixed model estimates. The code provides access to initialise parameters $p_{0}, d_{max}, p_{psc}, EC_{50}, g_{age}, p_{age}$ and fix parameters $N, C_{0}, k, r_{0}, \Delta r, A_{out}, d_{const}, L, s, \mu, \Delta t, r_{min}, t_{interval}$. Note, $p_{psc}$ represents the model parameter $p_{MCC}$.

## Description of Pancreas.hpp

In this script, the different aspects of the pancreas (tumour and surrounding healthy tissue) are defined. In this script is also the definition for SimulateOneDay().

double SimulateOneDay(…): Given some day and pancreas object, this function will simulate the model forward by running a for loop that simulates the model forward an hour at a time. This script is where cell neighbours are described, the pancreas object is initialised, cell locations are definitely and the model function iterating the model further each hour is defined.

## Description of Cell.hpp

In this script, we define action of cells. This includes cell death, cell proliferation, and cell movement. This includes defining cell states of Cancer = 1, Dead = 3, Healthy = 4, Empty = 5, PSC = 51.

## Description of Helper.hpp

In this script, we define some mathematical calculations to support the distance and neighbour calculations in the VCBM-PDE. This includes a function to define squaring a number Sqr(x) and the distance between two points ($X_{1}$,$Y_{1}$) and ($X_{2}$,$Y_{2}$): DistanceSquared(X1,Y1,X2,Y2).

## Description of delaunator.hpp

In this script, we calculate the Delaunay triangulation for a given lattice. This code was based on a JavaScript library for Delaunay triangulation of 2D points that can be found here <https://github.com/mapbox/delaunator>. A C# (https://github.com/nol1fe/delaunator-sharp) and C++ (https://github.com/delfrrr/delaunator-cpp) port of this JavaScript was used as inspiration for the delaunator.hpp script, which was written independent on these version.

## Description of Diffuse.hpp

In this script, we calculate the concentration of drug at specific points on the domain using a finite volume approximation. The ordinary differential equations (ODEs) are solved in two ways, either through an adaptive time-stepping process, derived based on MATLAB’s ode45 or through standard forward difference methods.


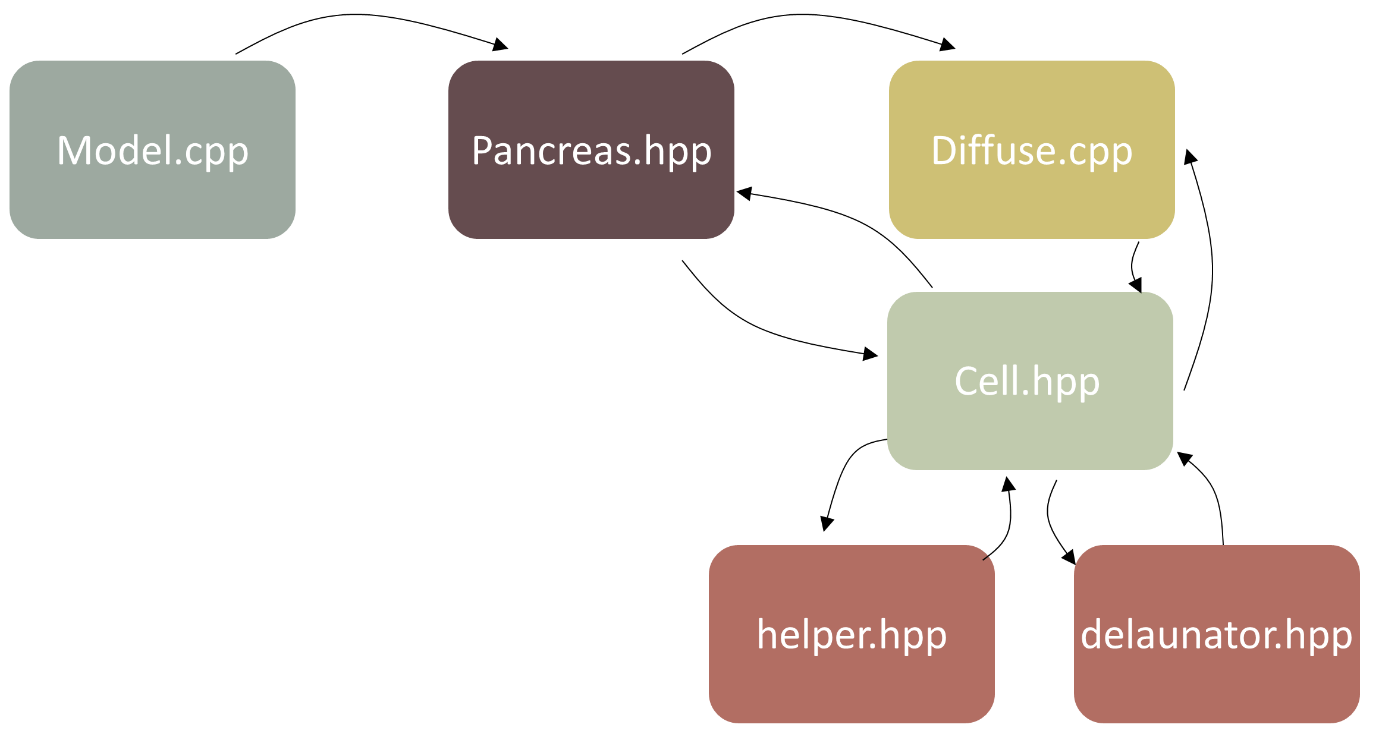


**Fig 1. Network structure of code for VCBM-PDE.** The schematic depicts how the different C++ scripts integrate together and their dependence.

# Cell agents

Each agent in the model is defined as a class Cells. Each Cell has the following major parts:

1. struct CellState: the current state of the cell in timestep $\Delta t$. This includes CellType, age, spring_length, sibling and X, Y position.
2. enum class CellType type: the phenotype of the cell represented by an integer value. Cancer = 1, Dead = 3, Healthy = 4, Empty = 5, PSC = 51.
3. int age: the age of the cell, i.e. the time spent in cell cycle.
4. double spring_length: the equilibrium spring length for that cell’s phenotype, $s$.
5. cell* sibling: pointer to sibling of that particular cell. When a cell divides into two cells, those cells become siblings.
6. double X, Y: $(x,y)$ position in the domain for the cell. Cell’s are represented by single $(x,y)$ points.
7. Vector<Cell*> Neighbours: vector list of points to an individual cell’s neighbours. Neighbours are cells that are directly linked to that cell using a Delaunay triangulation.

Key functions associated with Cell and located in Cell.hpp are detailed below:

1. void Renew(): sets new state to the current state.
2. void UpdateState():sets current state to the new state and increases cell age.
3. void Infect(): sets current cell to be a cancer cell.
4. void updateSibling(map<Cell*, Cell*> &map): updates cell siblings to correct sibling if cell has divided.
5. void clearNeighbours(): clears list of cell neighbours so new neighbour list can be defined.
6. bool onBoundary(): determines whether current cell is a Healthy cell and if it is on the boundary.
7. bool TooCrowded(): determines whether current cell has a neighbour within distance threshold, i.e. Sqr(Params::rmin).
8. double DistanceSquaredTo(Cell* cell): determines Euclidian distance between the current cell and another cell Cell* cell.
9. double DistanceSquaredFromCentre(): determines Euclidian distance between the cell and the centre of the tumour.
10. double DistanceFromBoundary(vector<Cell*> &boundaryCells): determines the distance between the current cell and those on the boundary, which are listed by vector<Cell*> &boundaryCells. The function finds the minimum Euclidian distance between the cell and the closest cell on the boundary/periphery of the tumour.
11. bool Necrotic(double distanceToBoundary, Params* parameters): determines whether the current cell is necrotic. Cell is classified as Necrotic if the distance to the boundary of the tumour is greater than or equal to $d_{max}$.
12. bool TooYoung(Params* parameters): determines whether a cell is too young to proliferate by comparing it’s age to $g_{age}$.
13. void LengthenSpring(Params* parameters): if the current cell $k$ has a sibling $i$ with a spring length less than $s$, then this function increases the spring length by $s_{k,i}+s/p_{age}$. Once a cell and it’s sibling have reached a spring length of $s$ then the sibling and the cell no longer track each other.
14. bool DrugInducedDeath(Params* parameters, double* drugConcentration, int gridRadius): determines whether cell has undergone drug-induced apoptosis based on the local concentration of drug double* drugConcentration.
15. void Die():if cell has died, update CellType to Dead and the age to 0.
16. void Disintegrate(): for dead cells, reduce the spring length by $s/8$ for 3 time steps, after which, set CellType to Empty.
17. void PossiblyPSCInfectNeighbour(Params* parameters): determines whether a new $p_{sc}$ cell was created in the given time-step.
18. void Move(): determines new position of the current cell using Hooke’s Law.
19. Cell* PossiblyPoliferate(vector<Cell*> &boundaryCells, Params* parameters): determines whether the cell proliferates by first checking TooYoung(), and TooCrowded(). It then uses DistanceFromBoundary(boundaryCells) to calculate the probability the cell proliferates.
20. Cell* Proliferate(Params* parameters): if the current cell proliferates, then this function determines the new positions of the new cells and also generates an associated age from a Poisson distribution.

# Diffusion script

We used a finite volume approximation for the PDE model of drug diffusion in the tumour, release from polymeric fibre, decay and uptake by cells. The full model equations are detailed in **S1 Technical** **Supplementary Information** and the solver script can be found in Diffuse.hpp. The main functions of this script are detailed below. These are based on MATLAB’s ode45.

1. void DiffuseSimple(double t0, double tfinal, double* y, int grid_size, int fibreX, int fibreY): takes in the time span to iterate on [t0, tfinal], i.e. start time t0 and final time tfinal, the drug concentration in the domain (outside the fibre) and inside the fibre in one vector y, the grid size and fibre positions. This function is the main function for solving the system of ODEs.
2. void odeFcn_main(double t, int G, double* y, double* dydt, int fibreX, int fibreY): takes in the value of the variables in the ode and simulates forward some time step $\Delta t$.

# Delaunay triangulation

A Delaunay triangulation is used to calculate the nearest neighbours for a cell agent in the simulation with script delaunator.hpp. This script was based of a JavaScript that can be found here (<https://github.com/mapbox/delaunator>). We do not go through the individual functions in this script as information on these can be found in the original JavaScript or C++ and C# ports (see <https://github.com/nol1fe/delaunator-sharp> and <https://github.com/delfrrr/delaunator-cpp>).

# Pancreas

The code Pancreas.cpp is the core script for the model and simulates the VCBM-PDE model forward by calling Cell.cpp and Diffuse.cpp. Each Pancreas has the following major parts:

class Pancreas: the pancreas class contains the cell and drug positions in a particular time step. It takes in parameter values Params* parameters including the drug injection location fibreX and fibreY. It also generates the map for cells in the VCBM.

1. vector<Cell*> cells: vector containing all cells in the simulation.
2. Params* parameters: parameters used in the simulation, either fixed in Params.hpp or initialised when the simulation was initially called.
3. vector<Cell*> new_cells: vector containing all the new cells added in a timestep
4. vector<Cell*> boundaryCells: vector containing all the boundary cells in the simulation
5. Cell* src, *dst: these are the labels for the two cells that are furthest apart on the tumour. This is used to calculate the approximate tumour volume.
6. int fibreX, fibreY: (X,Y) location for the injected drug or fibre.
7. long timeSinceInjection: tracking the time since the injection occurred with units minutes.
8. double drugConcentration[gridWidth * gridWidth + Params::N]: the drug concentration from a single injection.
9. double* fibreConcentration = drugConcentration +gridWidth * gridWidth: the drug concentration from an implanted fibre.

The key functions in this script and their associated actions are detailed below:

1. void InjectPoint(int x, int y, double amount): injects a concentration of drug amount into the location x, y in the domain.
2. void InjectFibre(int x, int y, double amount): injects a fibre with a concentration of drug amount into the location x,y in the domain. The fibre starts at location x,y and extends horizontally to the right.
3. double* LoadCellsCoordinates(): in each iteration of the model, this loads the location of cells after a single time step. It takes the positions X,Y from currentState.
4. void DetermineNeighbours(): determines the neighbours for a cell using the Delaunay triangulation and storing these in the Cell class member function Neighbours.
5. bool HealthyCellsBeyondRadius(double radius): checks whether there are any cells that are outside a distance radius from the centre of the tumour.
6. void AddNewCell(Cell* new_cell): adds a new cell to the cell class.
7. void AddMoreTissue(double moving_rim, double max_tumour_radius): adds more tissue onto the domain in an annulus of moving_rim. This tissue is made up of healthy cells which are roughly places into a hexagonal lattice.
8. void MoreTissueAddedIfNecessary(): if HealthyCellsBeyondRadius(double radius) is true then an additional rim of tissue is added to the outside of the domain by calling AddMoreTissue(…).
9. void DetermineBoundaryCells(): determines which cells are on the boundary of the tumour, i.e. have neighbouring cells as healthy cells. These cells are then stored in vector<Cell*> boundaryCells.
10. double TumourRadius(): calculates the tumour radius by finding the maximum distance between tumour cells.
11. double DistancetoLine(Cell* cell): calculates the distance of cell to the line joining the two cells that are furthest apart in the tumour domain Cell* src, *dst.
12. double TumourVolume(): calculates the tumour volume using Cell* src, *dst and the cell with the maximum DistancetoLine(Cell* cell) value.
13. double getPscRatio(): returns the ratio of the total number of cancer cells to psc cells.
14. CreateInitialTumour(): creates an initial lattice of healthy cells and initialises the cell in the centre as a cancer cell using Infect().
15. void SimulateOneHour(): simulates the VCBM-PDE forward by one hour. This first updates the drug concentration using DiffuseSimple, then updates the cell rules for each cell. The order of the algorithm is:

if (cell->OnBoundary())

cell->PossiblyPSCInfectNeighbour(parameters);

if (cell->currentState.type == CellType::Healthy)

cell->Move;

else if (cell->currentState.type == CellType::Dead)

cell->Disintegrate();

else if (cell->currentState.type == CellType::Empty)

{ }

else

{

if (cell->DrugInducedDeath(parameters, drugConcentration, gridRadius))

cell->Die();

else

{

if (cell->currentState.age < parameters->gage)

cell->LengthSpring(parameters);

Cell* newCell=cell->PossiblyProliferate(boundaryCells, parameters);

If (newCell != NULL)

AddNewCell(newCell);

Else

Cell->Move();

}

}

1. double SimulateOneDay(int day, void (*render)(int, int, Pancreas*, int)): simulates the VCBM-PDE forward one day by looping through SimulateOneHour().
2. int ReturnTotalNumberTumourCells(): returns the total number of tumour cells in the domain.
3. int ReturnTotalNumberDeadCells(): retuns the total number of dead cells in the domain.
4. int ReturnTotalNumberPSCCells(): returns the total number of PSC cells in the domain.
5. int ReturnTotalNumberHealthyCells(): returns the total number of Healthy cells in the domain.
6. double ReturnDrugConcentrationDomain(): returns the total drug concentration in the domain.
7. double ReturnDrugConcentrationinFibre(): returns the total drug concentration in the fibre
8. double ReturnDrugConcentrationAout(): returns the total drug concentration outsider the fibre.

# Plotting

The time-series output of the VCBM-PDE can either be plotted through Matlab or Visual studio (or another C++ program).

## Plotting in MATLAB

To plot in MATLAB, we use MATLAB’s inbuilt Voronoi tessellation voronoin which returns the Voronoi vertices and cells for a Voronoi diagram. The MATLAB script domain_plotter.m is used to plot the cells. This relies on calling ReturnNumberCells() and ReturnCellPositions(int index) and ReturnCellType(int index) which returns the number of cells in a simulation on a particular day, the $(x,y)$ position of a cell with index index and also it’s type.

## Plotting in C++

There are inbuilt functions in the C++ script, particularly void EnumerateVoronoiCells(…) and void EnumerateNeighbours(…) that allow you to create a plot of the VCBM-PDE in C++, however, we do not include instructions for these here.

# References

1. Jenner AL, Frascoli F, Coster ACF, Kim PS. Enhancing oncolytic virotherapy: Observations from a Voronoi Cell-Based model. J Theor Biol. 2020;485.
